# Evaluating Certainty of Evidence Assessment in Systematic Reviews of Yoga for Health: A Meta‐Research Study

**DOI:** 10.1002/cesm.70110

**Published:** 2026-10-31

**Authors:** Martial Sonkoue Pianta, Esra Abdalla, Yehuda Tri Nugroho Supranoto, Omolola Titilayo Alade, L. Susan Wieland

**Affiliations:** ^1^ Faculty of Health Sciences The University of Bamenda Bambili Cameroon; ^2^ Faculty of Medicine University of Khartoum Khartoum Sudan; ^3^ Harvard Medical School Boston Massachusetts USA; ^4^ Global Health Program, Faculty of Health Sciences McMaster University Hamilton Canada; ^5^ Georgetown University School of Medicine Washington District of Columbia USA

**Keywords:** certainty of evidence, yoga

## Abstract

**Background:**

The assessment of the trustworthiness and relevance of available evidence is a key element of evidence synthesis. For example, Cochrane systematic reviews (SRs) mandate the assessment of certainty of evidence (CoE) using the Grading of Recommendations, Assessment, Development and Evaluation (GRADE) method. However, most SRs are published outside Cochrane, and the use of CoE outside Cochrane is not universal. The use of CoE in SRs of yoga for maintaining or improving health is not known.

**Objective:**

To: (a) estimate the proportion of SRs of yoga for health that assessed CoE; (b) identify which CoE systems and domains were used; and (c) examine whether CoE assessment was associated with SR publication and methodological characteristics.

**Methods:**

The Cochrane Database of Systematic Reviews (CDSR), MEDLINE (Ovid), PubMed, EMBASE (Ovid), AMED (Ovid), CINAHL (EBSCO), and PsycINFO (EBSCO) were searched to April 25th 2024 for SRs of randomized controlled trials (RCTs) of yoga for health. Two reviewers independently screened studies against predefined eligibility criteria for SRs of yoga: the study (1) sought to comprehensively identify relevant RCTs on a yoga‐focused research question, (2) stated inclusion criteria for the studies, (3) searched at least one electronic database, and (4) reported the critical appraisal of the included studies. Data on SR characteristics and CoE were independently extracted from included SRs by two authors. Data analysis included frequencies and univariate and multivariable logistic regressions, conducted using R version 4.5.2. Statistical significance was set at *p* < .05.

**Results:**

A total of 250 SRs published between 2005 and 2025 were included. More than a third of the SRs (88/250; 35.2%) were published by authors based in Asia, America and Europe. Among the 246 reviews with included RCTs, 39 (16%, 95% CI = 11.8%–20.9%) assessed CoE for at least one effect estimate. Among reviews that assessed CoE, GRADE was the most frequently used framework (35/39, 89.7%). In multivariable analysis, CoE assessment was independently associated with presence of a meta‐analysis (OR = 3.01, 95% CI = 1.08–10.21), systematic‐review journal type (OR = 56.47, 95% CI = 5.33–1151.95), and use of a Cochrane risk‐of‐bias tool (OR = 12.17, 95% CI = 2.71–124.93). Region, journal impact factor, and study types included were not independently associated with CoE assessment after adjustment.

**Conclusion:**

Approximately 16% of SRs of yoga for health assessed CoE. Since this study was limited to English‐language studies identified from major databases through 2024, we cannot be certain whether this result reflects true levels of CoE assessment across all SRs of yoga for health conducted to date. Strengthening reviewer training, instituting journal requirements, and encouraging adherence to PRISMA and GRADE guidelines might increase the future percentage of yoga SRs assessing CoE.

## 1 Background

Yoga's popularity has surged globally as both a therapeutic and preventive intervention for conditions such as cardiovascular disease, metabolic disorders, cancer and depression [1, 2, 3]. In the United States, adult yoga practice rose from about 5.0% in 2002 to roughly 16.8% by 2022, this represents a tripling of yoga adoption in US adults over two decades [4]. Similar upward trends have been reported internationally, with hundreds of millions of people now practicing yoga worldwide [5]. This surge in popularity has been mirrored by a substantial growth in the research base [6, 7, 8, 9]. A bibliometric analysis identified 322 systematic reviews of yoga for health published up to 2019, with 81% published in 2012 or later [9].

SRs of randomized controlled trials (RCTs) are designed to gather, appraise, and synthesize evidence addressing a focused research question using prespecified, reproducible, and transparent methods. When conducted and reported rigorously, they are considered among the most reliable sources of evidence for clinical practice and guideline development. However, the ability of readers to understand, trust, and use the findings from an SR depends on how clearly authors evaluate and communicate the certainty of the body of evidence (CoE) supporting their conclusions.

CoE reflects the degree of confidence that an observed effect estimate approximates the true effect and is therefore central to judging the trustworthiness and applicability of review findings. Several features of the yoga RCT literature complicate the interpretation of pooled effects. A recent bibliometric analysis of yoga RCTs reported a median sample size of only 59 participants, with substantial heterogeneity in participant characteristics, yoga styles, intervention duration, comparator types, and outcome measures [6]. Such variability can contribute to imprecision, inconsistency, and indirectness, all of which reduce confidence in effect estimates. Therefore, SRs must assess not just the direction and size of effects, and the general quality of the included evidence, but also how confident one can be that these estimates correspond to true underlying effects.

Among the available approaches to assessing evidence certainty, GRADE (Grading of Recommendations, Assessment, Development and Evaluation) is a prominent framework for evaluating the certainty of evidence in SRs. GRADE evaluates domains such as risk of bias, inconsistency, indirectness, imprecision, and publication bias to classify the certainty of evidence from SRs of RCTs as high, moderate, low, or very low [10]. Organizations such as Cochrane and the Agency for Healthcare Research and Quality (AHRQ) use GRADE in reporting of evidence syntheses, and GRADE is also sometimes used in journal publications of evidence syntheses. Clear reporting of CoE and the underlying judgments supporting CoE helps readers interpret effect estimates in the context of their credibility in addressing the review question, supporting more informed clinical, research, and policy decisions.

Despite the substantial growth in yoga SRs of RCTs, it remains unclear to what extent authors assess or report CoE, which approaches are used, and whether reporting varies according to review characteristics. To address these gaps, the objective of this methods study was to examine the use of CoE assessments in SRs of RCTs of yoga interventions for health. Specifically, we aimed to [1]: estimate the proportion of SRs of yoga for health that assess CoE [2]; identify which CoE systems and domains were used; and [3] examine whether CoE assessment was associated with SR publication and methodological characteristics.

## 2 Methods

This is a cross‐sectional meta‐research study of published SRs.

### 2.1 Review Question

In SRs of RCTs of yoga for health, what is the prevalence and characteristics of CoE assessment?

The primary outcome for this study is the prevalence of CoE assessment. Secondary outcomes include the CoE systems reported to be used, the CoE domains reported to be used, and the associations of CoE assessment with SR publication and methodological characteristics.

### 2.2 Eligibility Criteria

We included completed SRs of RCTs in which yoga was explicitly identified as the primary intervention, excluding studies where yoga appeared only as a search term or secondary component (e.g., within broader reviews of exercise therapy). We included SRs that (1) sought to comprehensively identify relevant RCTs on a yoga‐focused research question, (2) stated inclusion criteria for the studies, (3) searched at least one electronic database, and (4) reported the critical appraisal of the included studies. The first criterion is to establish that the SR target was RCTs of yoga. The second through fourth criteria are methodological requirements for the studies to be considered SRs.

Eligible articles were required to have an SR as their main objective; guideline papers or other documents that conducted a review solely as a component of a larger project were excluded. We also excluded overviews, umbrella reviews, and scoping reviews. We excluded reviews that called themselves narrative reviews, literature reviews, or integrative reviews unless the title or abstract clearly indicated that SR methods were used. Although we originally planned to include studies in all languages, we excluded non‐English studies for practical reasons. There was no restriction on publication date or publication type, except that SRs available only as conference abstracts were excluded due to insufficient methodological detail. For SRs with multiple associated records, the most recent journal publication for the primary SR question was considered the primary record.

### 2.3 Study Protocol and Deviations

The study protocol was uploaded to the Open Science Framework (https://osf.io/8g7zt/files/6n38b). There are some deviations from the original protocol: First, we originally planned to include studies without regard to language of publication, secondly, we originally planned to focus on CoE for both RCT and non‐RCT evidence. Due to limited time and a lack of project funding, we decided at the initiation of screening to include only English‐language study reports, and to examine only CoE assessment of RCT evidence. Thirdly, we initially planned to use chi‐square tests to examine the association between variables but we decided to use logistic regression instead as a more robust method of analysis.

### 2.4 Study Reporting Framework

Although this is not an intervention SR, the literature search, screening, and selection processes followed systematic methods and are reported in accordance with relevant PRISMA 2020 items [11]. This approach is consistent with previous meta‐research studies that have used the PRISMA framework to report the systematic identification and selection of studies [12, 13].

### 2.5 Search and Selection Process

We used a database that had been developed for a previous study of SRs on yoga as a health intervention, which had conducted searches in the Cochrane Database of Systematic Reviews (CDSR), MEDLINE (Ovid), PubMed, EMBASE (Ovid), AMED (Ovid), CINAHL (EBSCO), and PsycINFO (EBSCO) [9]. An information specialist updated the searches to identify newly published SRs. The full strategy for the updated search is provided in Appendix 1. All records from the updated search were imported into EndNote and deduplicated, then imported into Covidence for screening together with the records from the previous study. Since the inclusion criteria for the original bibliometric analysis were broader than the inclusion criteria for the current study, two authors (MSP, EA) independently screened the title and abstract of all previous and newly identified records against the inclusion criteria for the current study. We obtained the full texts of records potentially meeting the eligibility criteria and independently screened them for inclusion in the review. Disagreements were resolved through consultation with a third reviewer (LSW).

### 2.6 Data Extraction

Two authors (L.S.W. and M.S.P.) designed, piloted, and refined a data extraction form. Two authors (M.S.P. and E.A.) independently extracted data using the finalized form, and any discrepancies were resolved through discussion or consultation with a third reviewer (L.S.W.). We did not contact SR authors for missing information.

The primary outcome was the prevalence of CoE assessment for RCT evidence. We recorded whether CoE was assessed, based on the report of the study authors. We only considered CoE to be assessed when the authors stated that they assessed the quality or certainty of the evidence for an effect estimate or result based on RCTs. When the authors stated that they used a CoE framework to assess the risk of bias or methodological quality of individual studies or the review as a whole, we noted this as a mention of CoE assessment, but we did not consider it to be a true CoE assessment.

We also extracted the CoE assessment system stated to be used, and the domains reported as evaluated for the CoE assessment. In our extraction of CoE domains, we relied upon the description of the authors regarding the domains that were considered. In cases where the authors did not explicitly list domains in the text but did present tables or descriptions of CoE assessments, we checked those for specific CoE domains referenced.

To characterize the SRs and analyze associations between review characteristics and CoE assessment, we extracted the following data: first author, year of publication, number of authors, journal name and type, journal impact factor, country of the corresponding author, health condition, type of funder, study types eligible for inclusion (RCTs only or RCTs plus non‐RCTs), number of included RCTs, risk‐of‐bias tools used for RCTs, and whether a meta‐analysis was performed. Extracted variables were selected as factors potentially influencing CoE assessment, based on previous meta‐research/meta‐epidemiological studies [14, 15, 16]. Number of authors was used as an indicator of research team capacity, journal characteristics (impact factor, journal type) were used as an indicator of editorial rigor, and funding type was used as an indicator of institutional and methodological support. We included country of the corresponding author, health condition, number of included RCTs, and eligibility of non‐RCTs as additional potentially exploratory variables.

### 2.7 Data Synthesis and Analysis

Data were analysed with R version 4.5.2. Study characteristics were summarized using descriptive statistics, reported as frequencies and percentages for categorical variables and as median (interquartile range) for year of publication and journal impact factor. The study characteristics examined included region of the corresponding author, year of publication, number of authors, journal impact factor, journal type, type of funder, type of eligible studies (RCTs only vs. RCTs plus other designs), presence of a meta‐analysis, type of health condition, and use of a Cochrane risk‐of‐bias tool. Country was aggregated into five geographic regions, and type of health condition was recoded from free text into seven clinical domains, both to avoid small subgroup sizes. The journal impact factor for non‐indexed journals was coded as missing and ambiguous characteristics were coded as “Unclear.” Associations between CoE assessment (our primary outcome) and study characteristics (potential predictors) were evaluated using univariable logistic regression, reported as odds ratios with 95% confidence intervals and statistical significance was set at *p* < .05. A multivariable model including all variables significant at the univariable level was fitted using Firth's penalized logistic regression to address quasi‐complete separation. No summary statistics were pooled across studies, consistent with the descriptive nature of this meta‐research study.

## 3 Results

A total of 3390 records were identified through database searches. After removal of 1624 duplicates (manual: *n* = 456; Covidence: *n* = 1168), 1,763 records were screened at title and abstract level and 317 reports were assessed in full‐text for eligibility. Sixty‐seven records were excluded for the following reasons: not meeting criteria for a systematic review of RCTs (*n* = 28); abstract or summary publication type (n = 18); intervention not restricted to yoga (*n* = 14); systematic review incorporated only as a component of a larger project (*n* = 4); not in English (*n* = 2); and not focused on a health condition or symptom (*n* = 1). A total of 250 SRs met the inclusion criteria. See Figure 1 for a PRISMA flowchart describing the screening and selection process, Appendix 2 for citations to excluded studies, and Appendix 3 for the citations to the included SRs.

**Figure 1:**
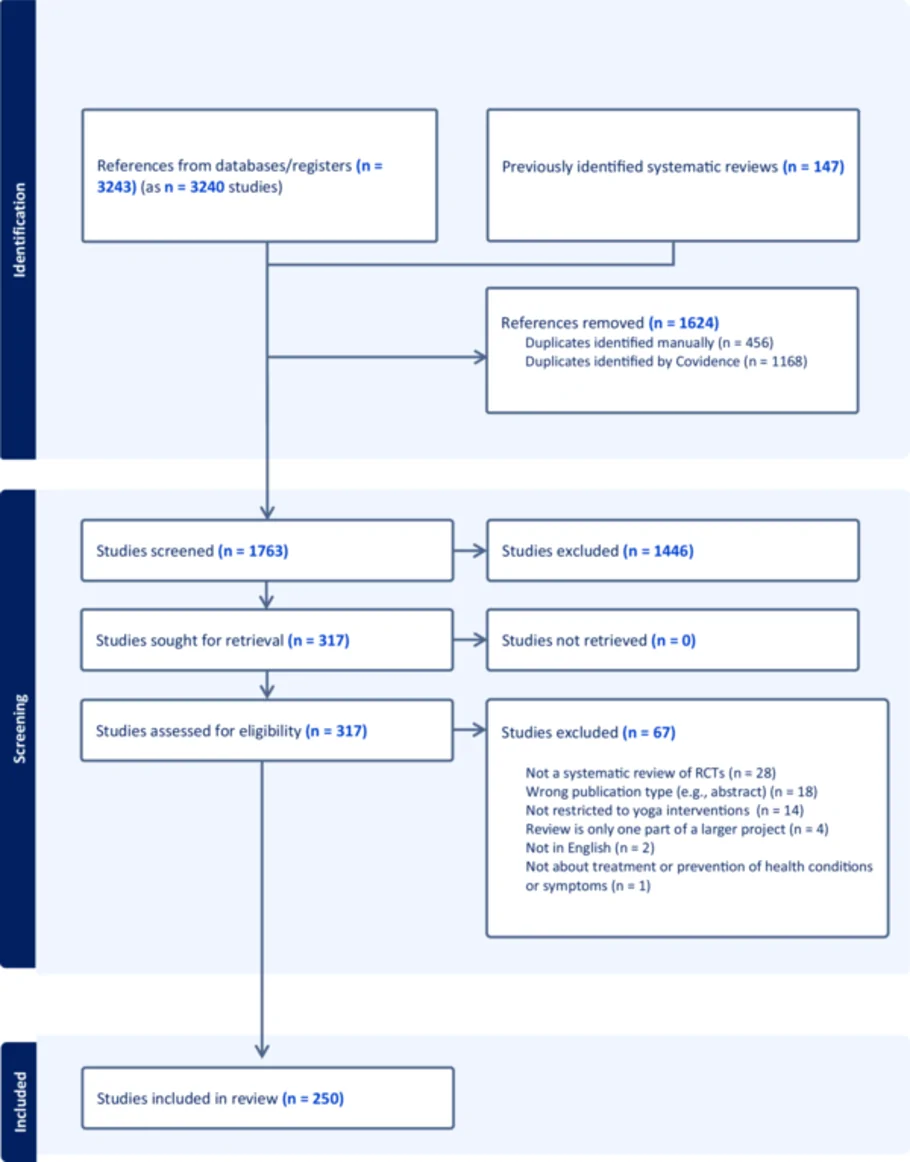
PRISMA flow chart of study selection.

### 3.1 Characteristics of Included Studies

The global regions with the most authors were Asia (primarily China), (88/250; 35.2%) followed by the Americas (primarily the USA) (72/250; 28.8%) and Europe (primarily Germany) (71/250; 28.4%). No included review had a corresponding author based in Africa.

Publication volume grew steadily over time, beginning with the first review in 2005 and accelerating between 2020 and 2024, when 42.4% of the studies (106/250) appeared.

The median number of authors was 4 (IQR 3, 6). The journal impact factor was missing for 33/250 SRs and for the remaining 217 SRs the median journal impact factor was 2.7 (IQR 2.1, 3.4). Specialty medical journals accounted for 52% of the publications (130/250), followed by complementary medicine journals at 30.8% (77/250).

In terms of financial support, 35% (88/250) of SRs reported government or non‐profit funding, whereas 31.6% (79/250) reported receiving no funding. Most SRs (147/250; 58.8%) restricted inclusion to RCTs and 145/250 (58%) conducted a meta‐analysis. After excluding four SRs with no included RCTs and three SRs with an unclear number of RCTs, the median number of included studies was 9 (IQR 5, 15). Cardiometabolic conditions represented the most studied health conditions, the focus of 18% of the reviews (45/250).

Several risk of bias assessment tools for RCTs were used in the SRs, though the original Cochrane Risk of Bias tool (Cochrane RoB1) was the predominant choice, used in 52.8% of cases (132/250). See Table 1 for SR characteristics.

**Table 1:** Characteristics of included reviews.

Characteristics	Summary statistic (*N* = 250)
**Region**	
Americas	72 (28.8%)
Asia	88 (35.2%)
Europe	71(28.4%)
Middle East	6 (2.4%)
Oceania	13 (5.2%)
**Year of publication, median (IQR)**	2019 (2015‐2021)
**Impact factor, median (IQR)**	2.7 (2.1‐3.4)
None	33 (13.2%)
**Number of authors**	
Five or more authors	117 (46.8%)
Fewer than five authors	133 (53.2%)
**Journal type**	
Complementary medicine	73 (29.2%)
General medical	19 (7.6%)
Specialty	132 (52.8%)
Systematic reviews	12 (4.8%)
Other	10 (4%)
**Type of funder**	
Government	53 (21.2%)
Government and Non‐profit	7(2.8%)
Non‐profit	28 (11.2%)
None (explicit)	79 (31.6%)
Unclear	83 (33.2%)
**Study types eligible for inclusion**	
RCTs and other study designs	103 (41.2%)
RCTs only	147 (58.8%)
**Presence of meta‐analysis**	
N/A (no included studies)	2 (0.8%)
No	103 (41.2%)
Yes	145 (58.0%)
**Type of health condition**	
Cancer	34 (13.6%)
Cardiometabolic	45 (18.0%)
Gynecology	22 (8.8%)
Muscoloskeletal	26 (10%)
Neurological/Cognitive	20 (8.0%)
Psychiatric/Psychological	43 (17.2%)
Other/Mixed/Multiple conditions	56 (22.4%)
ROB tools*	
Cochrane ROB1	132 (52.8%)
Cochrane ROB2	29 (11.6%)
Cochrane Back Review Group	7 (2.8%)
PEDro Scale	24 (9.6%)
Jadad scale	20 (8.0%)
Downs and Black	6 (2.4%)
CONSORT**	4 (1.6%)
Other	56 (22.4%)

*Note:* * More than one tool could be used. **CONSORT is a reporting guideline not a bias assessment, however it was explicitly used as a bias assessment tool in some reviews.

### 3.2 Proportion of SRS Assessing CoE

Removing the four SRs that did not include RCTs left 246 SRs eligible for evaluation of CoE; a denominator of 246 was therefore used for all descriptives and univariate and multivariable analyses. Among these 246 SRs, 41 reported assessing CoE for RCT evidence. However, two SRs (2/246; 0.8%) used the CoE framework to rate individual study quality rather than the certainty of evidence for an effect estimate. Overall, 16% (39/246, 95% CI: 11.8%–20.9%) of the eligible SRs evaluated CoE, while 84% (207/246, 95% CI: 79.1%–88.2%) did not.

### 3.3 CoE Assessment Tools and Domains Evaluated

Almost all SRs assessing CoE used GRADE (35/39; 90%). Other frameworks for CoE were the JBI levels of evidence and recommendations (*n* = 1), Cochrane Back review guidelines (*n* = 1), an unnamed tool (*n* = 1) and CoE assessment without apparent use of a structured tool (*n* = 1).

All 35 SRs that gave explicit criteria for rating CoE cited at least one of GRADE's five primary downgrading factors: risk of bias, inconsistency, indirectness, imprecision, or publication bias. Additional rating criteria reported were magnitude of effect (2/39), dose‐response gradient (1/39), residual confounding (1/39), and ‘other' risk of bias (4/39). (See Figure 2).

**Figure 2:**
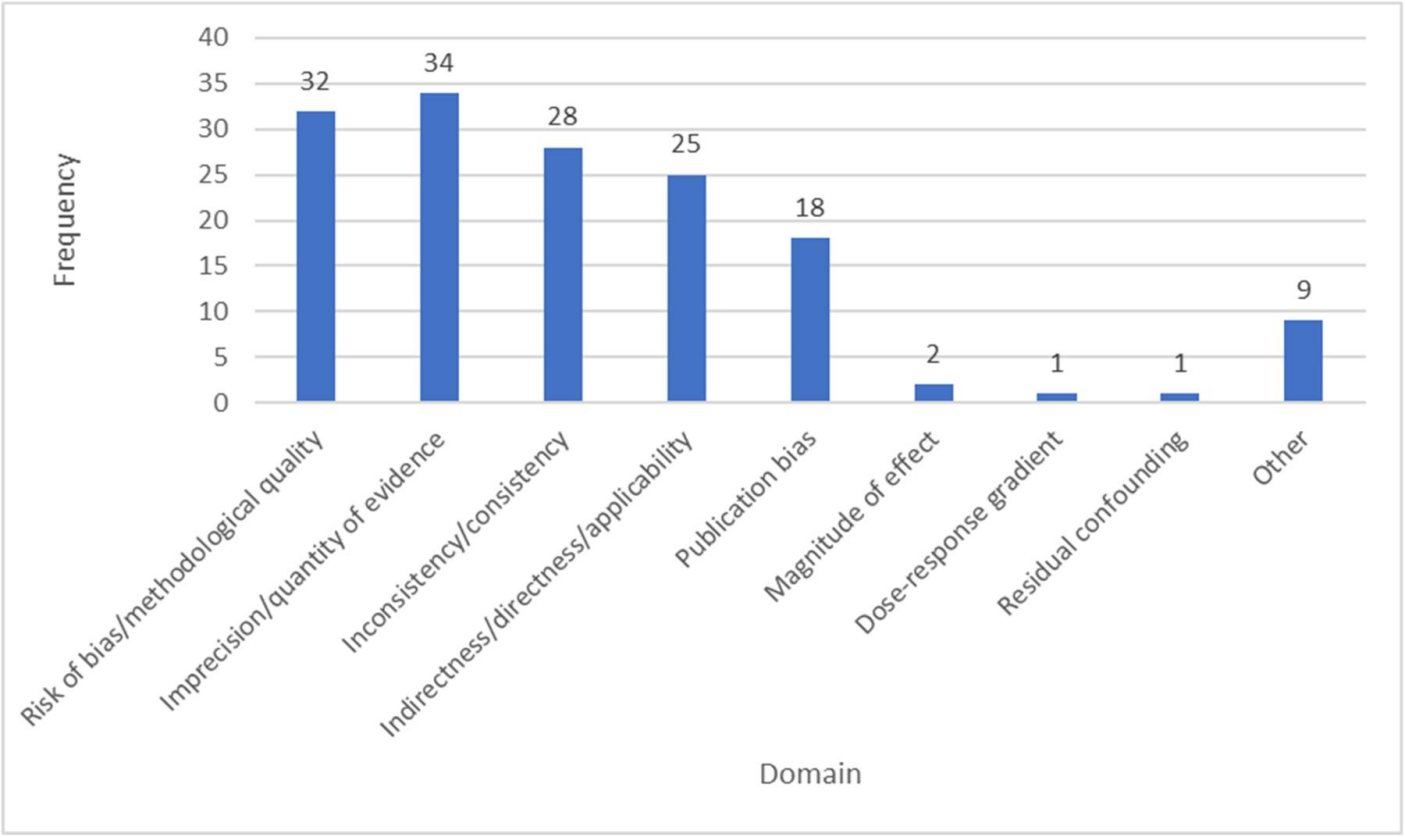
Frequency of reviews reporting the use of each domain in assessing CoE.

### 3.4 Trend in CoE Assessment Over Time and Predictors of Assessment

The first instance of CoE assessment was in 2012. While there was an increase in the publication of SRs of yoga for health over the past 20 years, the proportion of SRs assessing CoE did not increase in parallel (see Figure 3).

**Figure 3:**
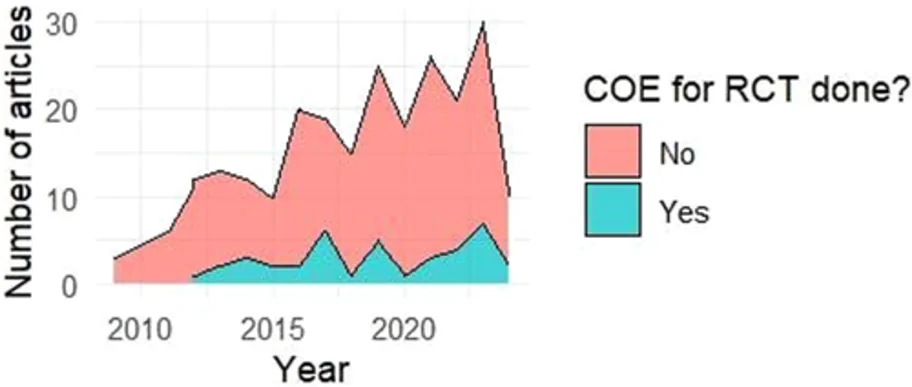
Trend in CoE assessment over time.

At the univariable level, CoE assessment was significantly associated with region (Europe vs Americas: OR = 3.08, 95% CI: 1.26–8.19, *p* = 0.013), journal impact factor (5_higher: OR = 6.69, 95% CI: 2.74–16.70, *p* < 0.001), journal type (systematic review journals: OR = 123, 95% CI 19.4–2456, *p* < 0.001), eligibility of RCTs only (OR = 2.67, 95% CI: 1.25–6.22, *p* = 0.015), presence of a meta‐analysis (OR = 4.67, 95% CI: 2.00–12.8, *p* < 0.001), and use of a Cochrane risk‐of‐bias tool (OR = 23.77, 95% CI: 3.85–977.54, *p* < 0.001). Year of publication, number of authors, type of funder, and type of health condition were not associated with CoE assessment (see Table 2).

**Table 2:** Association between characteristics of included studies and CoE assessment (Univariable logistic regression).

Characteristics	OR	95% CI	*p* value
**Region**			
Americas	—	—	—
Asia	1.49	0.58–4.07	0.417
Europe	3.08	1.26–8.19	0.013
Middle East	0.67	0.00–6.75	0.783
Oceania	1.90	0.32–8.31	0.440
**Year of publication**	1.06	0.96–1.16	0.185
**Number of authors**			
Fewer than five authors	—	—	—
Five or more authors	1.38	0.69–2.76	0.363
**Impact factor (JIF)**			
None–1.9	—	—	—
2–4.9	2.04	0.82–5.81	0.146
5–higher	13.6	4.64–16.70	< 0.001
**Journal type**			
Complementary medicine	—	—	—
General medical	1.31	0.18–6.13	.751
Specialty	1.88	0.75–5.37	0.201
Systematic reviews	123	19.4–2456	< 0.001
Other	1.24	0.06–8.51	0.850
**Type of funder**			
Government	—	—	—
Government and Non‐profit	0.22	0.00–2	0.212
Non‐profit	1.34	0.47–3.71	0.572
None (explicit)	0.50	0.20–1.25	0.138
Unclear	0.42	0.16–1.05	0.064
**Types of eligible studies**			
RCTs and other study designs	—	—	—
RCTs only	2.67	1.25–6.22	0.015
**Presence of meta‐analysis**			
No	—	—	—
Yes	4.67	2.00–12.8	< 0.001
**Type of health condition**			
Cancer	—	—	—
Cardiometabolic	1.07	0.31–3.93	0.917
Gynecology	0.92	0.17–4.19	0.911
Muscoloskeletal	1.38	0.34–5.56	0.642
Neurological/Cognitive	1.45	0.32–6.25	0.615
Psychiatric/Psychological	1.33	0.40–4.80	0.651
Other/Mixed/Multiple conditions	0.83	0.24–3.02	0.766
**Cochrane‐associated risk of bias tool**			
No	—	—	—
Yes	23.767	3.85–977.54	< 0.001

After inclusion of all variables in the multivariable model, only presence of a meta‐analysis (OR = 3.01, 95% CI: 1.08–10.21, *p* = 0.035), journal type (systematic‐review journals: OR = 56.47, 95% CI: 5.33–1151.95, *p* < 0.001), and use of a Cochrane risk‐of‐bias tool (OR = 12.17, 95% CI: 2.71–124.93, *p* < 0.001) remained independently associated with CoE assessment; region, journal impact factor and type of studies included were no longer significant, suggesting these univariable associations may be explained by their overlap with the three characteristics that remained significant (see Table 3).

**Table 3:** Association between characteristics of included studies and appropriate CoE assessment (multivariable logistic regression).

Variable	OR	95% CI	*p* value
**Region**			
Americas	—	—	—
Asia	1.14	0.35–4.03	0.831
Europe	1.37	0.4–4.99	0.617
Middle East	0.50	0–7.23	0.654
Oceania	1.49	0.21–8.59	0.666
**Types of studies included**			
RCTs and other study designs	—	—	—
RCTs only	0.65	0.24–1.76	0.390
**Presence of meta‐analysis**			
No	—	—	—
Yes	3.01	1.08–10.21	0.035
**Impact factor (JIF)**			
None–1.9	—	—	—
2–4.9	1.75	0.68–5.05	0.254
5–higher	2.43	0.47–12.13	0.281
**Type of journal**			
Complementary medicine	—	—	—
General medical	0.99	0.15–4.94	0.990
Specialty	1.45	0.52–4.48	0.485
Systematic reviews	56.47	5.33–1151.95	< 0.001
Other	0.86	0.08–5.34	0.878
**Cochrane‐associated risk of bias tool**			
No	—	—	—
Yes	12.17	2.71–124.93	< 0.001

## 4 Discussion

This study evaluated the use of CoE assessment in SRs of RCTs investigating yoga interventions for health. Among eligible reviews, 16% (39/246) conducted an outcome‐level certainty assessment, and 83.3% (205/246) did not. This proportion is lower than that reported by previous studies in fields such as sports medicine (20.6%), rehabilitation (29%), general medicine (33.8%), and traditional Chinese medicine (38.1%) [14, 15, 16, 17]. Several factors may explain the low level of CoE assessment. First, our comprehensive inclusion of all published SRs, whereas other studies focused on recent publications or select high‐impact journals, likely contributed to the lower observed percentage. Second, the extent to which authors of yoga SRs are familiar with contemporary SR methodology remains uncertain. More broadly, a general lack of familiarity with GRADE and alternative CoE frameworks, combined with their perceived complexity and inadequate formal training, continues to serve as a major barrier to widespread implementation among SR authors [18]. Journal submission requirements also appear to play a critical role, as large cross‐sectional analyses of journal author instructions demonstrate that fewer than 1% explicitly mention CoE assessment and that guidance on SR methodology is generally limited or absent, even among MEDLINE‐indexed journals [19, 20].

We found that GRADE was by far the most commonly used CoE framework among SRs that evaluated certainty, mirroring findings from similar studies in other fields [14, 15, 16]. This alignment can be explained by the worldwide adoption of the GRADE methodology over the last decade, with over 120 organizations endorsing or using GRADE (gradeworkinggroup.org). Review authors focused almost exclusively on GRADE's downgrading factors. Upgrading factors, such as large magnitude of effect, presence of a dose–response gradient, and residual confounding, were rarely mentioned. This aligns with standard GRADE practice for RCT evidence and is consistent with findings across general medicine [16], obstetrics and gynecology [21], urology [22], and nutrition [23].

A small proportion of SRs (0.8%) in our study used CoE inappropriately by applying the GRADE framework to rate the risk of bias of individual RCTs rather than to assess the certainty of the body of evidence for an effect estimate. GRADE is frequently misapplied, particularly by authors without formal training, resulting in misinterpretation of core constructs such as “quality,” “certainty,” and “grade.” [24] Importantly, our study did not evaluate the correctness or methodological rigor of CoE assessments, and therefore the overall quality of GRADE application within included SRs may have been suboptimal.

Although the publication of yoga‐related SRs increased steadily over the past decade, the frequency of CoE assessment did not increase proportionately. This contrasts with findings from sports medicine, where CoE assessment increased significantly over time, though that study evaluated SRs in high‐impact journals exclusively [15]. This indicates a need for researchers in the field of yoga/integrative medicine to adopt CoE assessment when conducting SRs.

Several characteristics were significantly associated with CoE assessment at the univariable level, including the country of the corresponding author, journal impact factor, journal type, type of included studies, presence of a meta‐analysis, and use of a Cochrane risk of bias tool. However, after adjustment in the multivariable model, only the presence of a meta‐analysis, journal type, and the use of a Cochrane risk of bias tool remained independently associated with CoE assessment (see Table 3).

The association between meta‐analysis and CoE rating aligns with previous findings in rehabilitation medicine [14]. Pooled effect estimates generated from meta‐analysis make CoE assessment more feasible by facilitating judgment across domains such as inconsistency, imprecision, and publication bias. Journal type was similarly predictive, with SR journals showing higher adoption compared to integrative medicine journals. While it is expected that evidence synthesis journals enforce strict adherence to SR standards, consistent CoE assessment must extend across all journal categories, given that yoga SRs are published and accessed across multiple journal types. Finally, SRs using a Cochrane risk of bias tool were significantly more likely to assess CoE. Risk of bias evaluations using Cochrane tools directly inform GRADE's core domains, and their application within SRs strengthens the credibility of recommendations [25]. The use of Cochrane tools may reflect greater author awareness of broader Cochrane standards for SR conduct; however, notably, even among reviews employing Cochrane risk of bias assessments, the majority still failed to evaluate certainty of evidence using GRADE.

### 4.1 Strengths and Limitations

This study provides a broad, contemporary evaluation of CoE assessment practices across two decades of SRs of yoga interventions for health. We summarized data across a large sample of SRs and offered a comprehensive mapping of how CoE assessment is applied in this field, highlighting key factors associated with its use. However, several limitations should be considered when interpreting these findings. First, our analyses relied strictly on information reported in published SRs. It's possible that some review authors may have evaluated CoE without reporting it, which could have resulted in underestimation of its use. However, because CoE assessment is expected to be explicitly reported to inform interpretation of review findings, we believe any such underestimation is likely to be limited.

Second, the scope of our evaluation was limited to determining the presence or absence of CoE assessment, and we did not attempt to assess the correctness of its application to effect estimates. Therefore, our CoE estimates likely overstate the extent of correct CoE application in this literature.

Third, language restriction, limited searching activities, and database coverage may limit generalizability to SRs published in other languages or in non‐indexed journals. We did not search gray literature sources, perform handsearching, or conduct citation chasing to identify additional SRs of yoga. Given that CoE assessment was associated with publication in higher‐impact journals, the omission of non‐indexed and non‐English literature may slightly overestimate the true extent of CoE assessment across the broader yoga literature.

Lastly, our study was restricted to SRs synthesizing RCTs. Consequently, our findings may not be generalizable to SRs predominantly including observational studies, where certainty assessment involves additional considerations, such as upgrading domains within the GRADE framework.

## 5 Conclusion

This meta‐research study examined the assessment of CoE in SRs of yoga interventions for health. Despite the growing number of yoga‐related SRs over the past two decades, only approximately one in six (39/246, 16.0%) conducted an outcome‐level CoE assessment for RCT evidence. Reviewers mainly relied on GRADE for CoE assessment, with core domains such as risk of bias, imprecision, and inconsistency applied most frequently. Outcome‐level certainty assessment was independently associated with conducting a meta‐analysis, publication in SR journals, and utilizing a Cochrane risk of bias tool. These associations suggest that author training on methods, journal editorial policy, and closer adherence to PRISMA and GRADE guidance may be relevant levers for improving CoE reporting. However, critical questions remain for future methodological research, including why CoE assessments remain infrequently applied, and whether CoE assessments are used appropriately when they are applied.

## Author Contributions

**Martial Sonkoue Pianta:** methodology, formal analysis, investigation, writing – original draft. **Esra Abdalla:** methodology, formal analysis, investigation, writing – original draft. **Yehuda Tri Nugroho Supranoto:** writing – review and editing. **Omolola Titilayo Alade:** writing – review and editing. **L. Susan Wieland:** conceptualization, methodology, data curation, writing – review and editing, supervision.

## Funding

The authors have nothing to report.

## Ethics Statement

This study is based on publicly available systematic reviews and does not involve human participants or identifiable personal data. Therefore, it was exempt from ethics committee approval.

## Conflicts of Interest

L.S.W. was the lead author on two of the included reviews; she was not involved in selection or data extraction for those reviews. L.S.W. has received grants, payments, travel and/or accommodation from governmental, non‐profit, and non‐governmental organizations for conducting research, presenting research, and providing expert advice about traditional, complementary and integrative medicine (TCIM) in general, but has no direct financial or non‐financial interests in the topic of this research. The other authors declare no conflicts of interest.

## Supporting information


Supporting File 1

Supporting File 2

Supporting File 3

Supporting File 4

## Data Availability

The authors confirm that the data supporting the findings of this study are available within its supporting materials.
