# Supplementary material for: Evaluating Certainty of Evidence Assessment in Systematic Reviews of Yoga for Health: A Meta‐Research Study
Source: Cochrane Evid Synth Methods. 2026 Oct 31;4(6):e70110. doi: 10.1002/cesm.70110 (PMC13723278; doi:10.1002/cesm.70110)
Supplement: Supplementary file 1 [file CESM-4-e70110-s004.docx]

**Appendix 1. Updated searches for yoga reviews**

**Searches carried out 25^th^ April 2024**

**PubMed**

Search: ((yoga[MeSH Terms]) OR (Yoga* OR Yogic OR Pranayam* OR Asana*)) AND ((systematic review* OR systematic overview OR metaanalysis OR meta-analysis OR systematic* review OR systematic[sb] OR meta-analysis[pt])) Filters: from 2019/11/23 - 3000/12/12.

Number of records: 623

**Medline (Ovid MEDLINE(R) Epub Ahead of Print, In-Process & Other Non-Indexed Citations, Ovid MEDLINE(R) Daily, Ovid MEDLINE and Versions(R)**

|  |  | Searches | Results | Type |
| --- | --- | --- | --- | --- |
|  | 1 | exp Yoga/ | 3987 | Advanced |
|  | 2 | (Yoga* or Yogic or Pranayam* or Asana*).mp. | 7998 | Advanced |
|  | 3 | 1 or 2 | 7998 | Advanced |
|  | 4 | (systematic review* or systematic overview or metaanalysis or meta-analysis or systematic* review or systematic or meta-analysis).mp. | 742182 | Advanced |
|  | 5 | 3 and 4 | 1137 | Advanced |
|  | 6 | 5 and 2019:2025.(sa_year). | 651 | Advanced |

**Embase (Ovid)**

|  | 1 | exp Yoga/ | 11821 | Advanced |
| --- | --- | --- | --- | --- |
|  | 2 | (Yoga* or Yogic or Pranayam* or Asana*).mp. | 14400 | Advanced |
|  | 3 | 1 or 2 | 14400 | Advanced |
|  | 4 | (systematic review* or systematic overview or metaanalysis or meta-analysis or systematic* review or systematic or meta-analysis).mp. | 1028872 | Advanced |
|  | 5 | 3 and 4 | 2160 | Advanced |
|  | 6 | 5 and 2019:2025.(sa_year). | 1212 | Advanced |

**AMED (Ovid)**

|  | 1 | exp Yoga/ | 1130 | Advanced |
| --- | --- | --- | --- | --- |
|  | 2 | (Yoga* or Yogic or Pranayam* or Asana*).mp. | 1343 | Advanced |
|  | 3 | 1 or 2 | 1343 | Advanced |
|  | 4 | (systematic review* or systematic overview or metaanalysis or meta-analysis or systematic* review or systematic or meta-analysis).mp. | 10800 | Advanced |
|  | 5 | 3 and 4 | 116 | Advanced |
|  | 6 | 5 and 2019:2025.(sa_year). | 60 | Advanced |

**Psycinfo (EBSCO)**

|  | S4 | **Limiters** - Publication Year: 2019-2024 | **Search modes** - Boolean/Phrase | 196 |
| --- | --- | --- | --- | --- |
|  | S3 | S1 AND S2 | **Search modes** - Boolean/Phrase | 391 |
|  | S2 | systematic review* OR systematic overview OR metaanalysis OR meta-analysis OR systematic* review OR systematic | **Search modes** - Boolean/Phrase | 148,781 |
|  | S1 | MW yoga OR ( Yoga* OR Yogic OR Pranayam* OR Asana* ) | **Search modes** - Boolean/Phrase | 3,970 |

**CINAHL (EBSCO)**

|  | S4 | **Limiters** - Publication Date: 20190101-20241231 | **Search modes** - Boolean/Phrase | (383) |
| --- | --- | --- | --- | --- |
|  | S3 | S1 AND S2 | **Search modes** - Boolean/Phrase | (767) |
|  | S2 | systematic review* OR systematic overview OR metaanalysis OR meta-analysis OR systematic* review OR systematic | **Search modes** - Boolean/Phrase | (297788) |
|  | S1 | MW yoga OR ( Yoga* OR Yogic OR Pranayam* OR Asana* ) | **Search modes** - Boolean/Phrase | (11789) |

**CDSR (Cochrane Database of Systematic Reviews)**

| #1 | MeSH descriptor: [Yoga] explode all trees | 1325 |
| --- | --- | --- |
| #2 | Yoga* or Yogic or Pranayam* or Asana* | 8255 |
| #3 | #1 or #2 | 8255 |

Limited to 23/11/2019 – 25/04/24; systematic reviews only

Results: 118 records (62 full reviews and 56 protocols)
