# Supplementary material for: Evaluating Certainty of Evidence Assessment in Systematic Reviews of Yoga for Health: A Meta‐Research Study
Source: Cochrane Evid Synth Methods. 2026 Oct 31;4(6):e70110. doi: 10.1002/cesm.70110 (PMC13723278; doi:10.1002/cesm.70110)
Supplement: Supplementary file 2 [file CESM-4-e70110-s001.doc]

Appendix 3: Included studies

1.	Abdelazeem B, Abbas K, Manasrah N, Amin M, Mohammed S, Mostafa M. Yoga as a treatment for vasovagal syncope: A systematic review and meta-analysis. Complementary Therapies in Clinical Practice. 2022;48.

2.	Ahuja N, Bhardwaj P, Pathania M, Sethi D, Kumar A, Parchani A, et al. Yoga Nidra for hypertension: A systematic review and meta-analysis. Journal of Ayurveda and integrative medicine. 2024;15(2):100882.

3.	Alexander G, Lockwood S. Effect of yoga on sleep quality among adult cancer patients: A systematic review. JBI Database System Rev Implement Rep. 2014;12(6):382-419.

4.	Ali M, Oble MJP, Sonia SN, George S, Shahi SR, Ali Z, et al. A Systematic Review of Quantitative and Qualitative Results of Randomized Controlled Trials Assessing the Effect of Yoga in Adult Women With Post-traumatic Stress Disorder: What Is Known So Far. Cureus. 2023;15(10):e47765.

5.	Aljasir B, Bryson M, Al-Shehri B. Yoga Practice for the Management of Type II Diabetes Mellitus in Adults: A systematic review. Evidence-based complementary and alternative medicine : eCAM. 2010;7(4):399-408.

6.	Anheyer D, Haller H, Lauche R, Dobos G, Cramer H. Yoga for treating low back pain: A systematic review and meta-analysis. Pain. 2022;163(4):E504-E17.

7.	Anheyer DKPLRSFJCH. Yoga for Treating Headaches: a Systematic Review and Meta-analysis. J Gen Intern Med. 2019;30:30.

8.	Anshu, Singh N, Deka S, Saraswati P, Sindhwani G, Goel A, et al. The effect of yoga on pulmonary function in patients with asthma: A meta-analysis. Complementary therapies in clinical practice. 2023;50:101682.

9.	Anuradha, Rojaria NS, Kaur J, Saini M. The Effects of Yoga on Cardiovascular Risk Factors among Patients with Type 2 Diabetes Mellitus: Systematic Review and Meta-Analysis. International journal of yoga. 2022;15(1):3-11.

10.	Armer JS, Lutgendorf SK. The Impact of Yoga on Fatigue in Cancer Survivorship: A Meta-Analysis. JNCI cancer spectrum. 2020;4(2):pkz098.

11.	Asiah ASS, Norhayati MN, Muhammad J, Muhamad R. Effect of yoga on anthropometry, quality of life, and lipid profile in patients with obesity and central obesity: A systematic review and meta-analysis. Complementary therapies in medicine. 2023;76:102959.

12.	Baishya A, Metri K. Effects of yoga on hypothyroidism: A systematic review. Journal of Ayurveda and integrative medicine. 2024;15(2):100891.

13.	Balasubramaniam M, Telles S, Doraiswamy PM. Yoga on our minds: A systematic review of yoga for neuropsychiatric disorders. Front Psychiatry. 2013;3 (JAN) (no pagination)(Article 117).

14.	Ban M, Yue X, Dou P, Zhang P. The Effects of Yoga on Patients with Parkinson's Disease: A Meta-Analysis of Randomized Controlled Trials. Behavioural Neurology. 2021;2021(no pagination)(5582488).

15.	Barrows JL, Fleury J. Systematic Review of Yoga Interventions to Promote Cardiovascular Health in Older Adults. Western journal of nursing research. 2016;38(6):753-81.

16.	Baydoun M, Oberoi D, Flynn M, Moran C, McLennan A, Piedalue KL, et al. Effects of Yoga-Based Interventions on Cancer-Associated Cognitive Decline: a Systematic Review. Current oncology reports. 2020;22(10):100.

17.	Belpu N, Yeshoda K. The effects of Yoga on voice: A systematic review. Journal of the All India Institute of Speech & Hearing. 2022;41(1):25-32.

18.	Bhagel P, Saha M. Effects of yogic intervention on pulmonary function and respiratory muscle strength parameters: A systematic literature review and meta-analysis. Journal of Biosciences. 2021;46(3) (no pagination)(76).

19.	Bhardwaj P, Ahuja N, Parchani A, Singh S, Sethi D, Pathania M. Yoga as a complementary therapy in neuropathic pain: A systematic review and meta-analysis of randomized controlled trials. Journal of family medicine and primary care. 2023;12(10):2214-22.

20.	Bhattacharyya KK, Andel R, Small BJ. Effects of yoga-related mind-body therapies on cognitive function in older adults: A systematic review with meta-analysis. Archives of gerontology and geriatrics. 2021;93:104319.

21.	Birdee GS, Yeh GY, Wayne PM, Phillips RS, Davis RB, Gardiner P. Clinical applications of yoga for the pediatric population: a systematic review. Academic pediatrics. 2009;9(4):212-20 e1.

22.	Boehm K, Ostermann T, Milazzo S, Bussing A. Effects of yoga interventions on fatigue: a meta-analysis. Evidence-based complementary and alternative medicine : eCAM. 2012;2012:124703.

23.	Boopalan D, Vijayakumar V, Ravi P, shanmugam P, Kunjumon B, Kuppusamy M. Effectiveness of antenatal yoga in reducing intensity of labour pain: A systematic review and meta-analysis. European Journal of Obstetrics and Gynecology and Reproductive Biology: X. 2023;19(no pagination)(100214).

24.	Borden A, Cook-Cottone C. Yoga and eating disorder prevention and treatment: A comprehensive review and meta-analysis. Eating disorders. 2020;28(4):400-37.

25.	Brandani JMJCEMH. The hypotensive effect of Yoga's breathing exercises: A systematic review. Complementary Therapies in Clinical Practice. 2017;28:38-46.

26.	Bressington DYCWWNTCCWT. The effects of group-based Laughter Yoga interventions on mental health in adults: A systematic review. J Psychiatr Ment Health Nurs. 2018;25(8):517-27.

27.	Bridges LSM. The Efficacy of Yoga as a Form of Treatment for Depression. J Evid Based Complementary Altern Med. 2017;22(4):1017-28.

28.	Brinsley J, Schuch F, Lederman O, Girard D, Smout M, Immink MA, et al. Effects of yoga on depressive symptoms in people with mental disorders: a systematic review and meta-analysis. British journal of sports medicine. 2021;55(17):992-1000.

29.	Broderick J, Knowles A, Chadwick J, Vancampfort D. Yoga versus standard care for schizophrenia. The Cochrane database of systematic reviews. 2015(10):CD010554.

30.	Broderick JCNWAVD. Yoga versus non-standard care for schizophrenia. Cochrane Database Syst Rev. 2017;9:CD012052.

31.	Broderick JVD. Yoga as part of a package of care versus standard care for schizophrenia. Cochrane Database Syst Rev. 2017;9:CD012145.

32.	Broderick JVD. Yoga as part of a package of care versus non-standard care for schizophrenia. Cochrane Database Syst Rev. 2019;4:CD012807.

33.	Brooks J, Lawlor S, Turetzkin S, Goodnight CW, Galantino ML. Yoga for Substance Use Disorder in Women: A Systematic Review. International journal of yoga therapy. 2021;31(1).

34.	Buffart LM, van Uffelen JG, Riphagen, II, Brug J, van Mechelen W, Brown WJ, et al. Physical and psychosocial benefits of yoga in cancer patients and survivors, a systematic review and meta-analysis of randomized controlled trials. BMC cancer. 2012;12:559.

35.	Bussing A, Ostermann T, Ludtke R, Michalsen A. Effects of yoga interventions on pain and pain-associated disability: a meta-analysis. The journal of pain : official journal of the American Pain Society. 2012;13(1):1-9.

36.	Caldwell AE, Purcell SA, Gray B, Smieja H, Catenacci VA. The impact of yoga on components of energy balance in adults with overweight or obesity: A systematic review. Obesity science & practice. 2022;8(2):219-32.

37.	Chandrababu R, Ramesh J, Jagadeesh NS, Guo P, Reddy GG, Hayter M. Effects of yoga on anxiety, pain, inflammatory and stress biomarkers in patients undergoing cardiac surgery: A systematic review and meta-analysis. Complementary therapies in clinical practice. 2023;53:101798.

38.	Chang DG, Holt JA, Sklar M, Groessl EJ. Yoga as a treatment for chronic low back pain: A systematic review of the literature. Journal of orthopedics & rheumatology. 2016;3(1):1-8.

39.	Chen S, Deng S, Liu Y, Yin T. Effects of Yoga on Blood Glucose and Lipid Profile of Type 2 Diabetes Patients Without Complications: A Systematic Review and Meta-Analysis. Frontiers in sports and active living. 2022;4:900815.

40.	Chen X, Zhen X, Zheng H, Chen H, Liao H, Zhu J, et al. Meta-analysis of effects of yoga exercise intervention on sleep quality in breast cancer patients. Frontiers in Oncology. 2023;13(no pagination)(1146433).

41.	Chetry D, Singh J, Chhetri A, Katiyar VK, Singh DS. Effect of yoga on intra-ocular pressure in patients with glaucoma: A systematic review and meta-analysis. Indian journal of ophthalmology. 2023;71(5):1757-65.

42.	Cheung C, Juyoung P, Wyman JF. Effects of Yoga on Symptoms, Physical Function, and Psychosocial Outcomes in Adults with Osteoarthritis. American Journal of Physical Medicine & Rehabilitation. 2016;95(2):139-51.

43.	Chia-Liang D, Sharma M, Ching-Chen C, Yesilyurt E, Godbey S. Yoga as an Alternative Therapy for Weight Management in Child and Adolescent Obesity: A Systematic Review and Implications for Research. Alternative Therapies in Health & Medicine. 2021;27(1):48-55.

44.	Chobe MP, Nanjundaiah RM, Chobe S, Raghuram N. Effect of yoga on adipokine levels among overweight and obese people: A systematic review. Journal of Ayurveda and Integrative Medicine. 2023;14(6) (no pagination)(100813).

45.	Chobe S, Chobe M, Metri K, Patra S, Nagaratna R. Impact of Yoga on cognition and mental health among elderly: A systematic review. Complementary Therapies in Medicine. 2020;52.

46.	Chong CS, Tsunaka M, Tsang HW, Chan EP, Cheung WM. Effects of yoga on stress management in healthy adults: A systematic review. Alternative therapies in health and medicine. 2011;17(1):32-8.

47.	Chu P, Gotink RA, Yeh GY, Goldie SJ, Hunink MG. The effectiveness of yoga in modifying risk factors for cardiovascular disease and metabolic syndrome: A systematic review and meta-analysis of randomized controlled trials. Eur J Prev Cardiolog. 2016;23(3):291-307.

48.	Chugh-Gupta N, Baldassarre FG, Vrkljan BH. A systematic review of yoga for state anxiety: considerations for occupational therapy. Canadian journal of occupational therapy Revue canadienne d'ergotherapie. 2013;80(3):150-70.

49.	Ciezar-Andersen SD, Hayden KA, King-Shier KM. A systematic review of yoga interventions for helping health professionals and students. Complementary therapies in medicine. 2021;58:102704.

50.	Cocchiara RA, Peruzzo M, Mannocci A, Ottolenghi L, Villari P, Polimeni A, et al. The use of yoga to manage stress and burnout in healthcare workers: A systematic review. Journal of Clinical Medicine. 2019;8(3) (no pagination)(284).

51.	Corrigan L, Moran P, McGrath N, Eustace-Cook J, Daly D. The characteristics and effectiveness of pregnancy yoga interventions: a systematic review and meta-analysis. BMC pregnancy and childbirth. 2022;22(1):250.

52.	Cramer H, Haller H, Klose P, Ward L, Chung VC, Lauche R. The risks and benefits of yoga for patients with chronic obstructive pulmonary disease: a systematic review and meta-analysis. Clinical rehabilitation. 2019;33(12):1847-62.

53.	Cramer H, Haller H, Lauche R, Steckhan N, Michalsen A, Dobos G. A systematic review and meta-analysis of yoga for hypertension. American journal of hypertension. 2014;27(9):1146-51.

54.	Cramer H, Lange S, Klose P, Paul A, Dobos G. Yoga for breast cancer patients and survivors: A systematic review and meta-analysis. BMC Cancer. 2012;12 (no pagination)(412).

55.	Cramer H, Lange S, Klose P, Paul A, Dobos G. Can yoga improve fatigue in breast cancer patients? A systematic review. Acta oncologica (Stockholm, Sweden). 2012;51(4):559-60.

56.	Cramer H, Langhorst J, Dobos G, Lauche R. Yoga for metabolic syndrome: A systematic review and meta-analysis. European journal of preventive cardiology. 2016.

57.	Cramer H, Lauche R, Azizi H, Dobos G, Langhorst J. Yoga for multiple sclerosis: a systematic review and meta-analysis. PloS one. 2014;9(11):e112414.

58.	Cramer H, Lauche R, Haller H, Dobos G. A systematic review and meta-analysis of yoga for low back pain. The Clinical journal of pain. 2013;29(5):450-60.

59.	Cramer H, Lauche R, Haller H, Dobos G, Michalsen A. A systematic review of yoga for heart disease. European journal of preventive cardiology. 2015;22(3):284-95.

60.	Cramer H, Lauche R, Haller H, Steckhan N, Michalsen A, Dobos G. Effects of yoga on cardiovascular disease risk factors: a systematic review and meta-analysis. International journal of cardiology. 2014;173(2):170-83.

61.	Cramer H, Lauche R, Klose P, Langhorst J, Dobos G. Yoga for schizophrenia: a systematic review and meta-analysis. BMC psychiatry. 2013;13:32.

62.	Cramer H, Lauche R, Langhorst J, Dobos G. Effectiveness of yoga for menopausal symptoms: a systematic review and meta-analysis of randomized controlled trials. Evidence-based complementary and alternative medicine : eCAM. 2012;2012:863905.

63.	Cramer H, Lauche R, Langhorst J, Dobos G. Yoga for depression: a systematic review and meta-analysis. Depression and anxiety. 2013;30(11):1068-83.

64.	Cramer H, Lauche R, Langhorst J, Dobos G. Yoga for rheumatic diseases: a systematic review. Rheumatology (Oxford, England). 2013;52(11):2025-30.

65.	Cramer H, Posadzki P, Dobos G, Langhorst J. Yoga for asthma: a systematic review and meta-analysis. Annals of allergy, asthma & immunology : official publication of the American College of Allergy, Asthma, & Immunology. 2014;112(6):503-10 e5.

66.	Cramer HADLRDG. A systematic review of yoga for major depressive disorder. J Affect Disord. 2017;213:70-7.

67.	Cramer HADSFJDG. Yoga for posttraumatic stress disorder - a systematic review and meta-analysis. BMC Psychiatry. 2018;18(1):72.

68.	Cramer HKPBBMADG. Effects of yoga on chronic neck pain: a systematic review and meta-analysis. Clin Rehabil. 2017;31(11):1457-65.

69.	Cramer HLRADPKdMMDGWL. Yoga for anxiety: A systematic review and meta-analysis of randomized controlled trials. Depress Anxiety. 2018;35(9):830-43.

70.	Cramer HLRKPLSLJDGJ. Yoga for improving health-related quality of life, mental health and cancer-related symptoms in women diagnosed with breast cancer. The Cochrane database of systematic reviews. 2017;1:CD010802.

71.	Cramer HPWLR. Yoga for menopausal symptoms-A systematic review and meta-analysis. Maturitas. 2018;109:13-25.

72.	Crow EM, Jeannot E, Trewhela A. Effectiveness of Iyengar yoga in treating spinal (back and neck) pain: A systematic review. International journal of yoga. 2015;8(1):3-14.

73.	Cui J, Yan JH, Yan LM, Pan L, Le JJ, Guo YZ. Effects of yoga in adults with type 2 diabetes mellitus: A meta-analysis. Journal of Diabetes Investigation. 2016.

74.	Curtis K, Weinrib A, Katz J. Systematic review of yoga for pregnant women: current status and future directions. Evidence-based complementary and alternative medicine : eCAM. 2012;2012:715942.

75.	Dai CL, Sharma M, Chen CC, Yesilyurt E, Godbey S. Yoga as an Alternative Therapy for Weight Management in Child and Adolescent Obesity: A Systematic Review and Implications for Research. Alternative therapies in health and medicine. 2021;27(1):48-55.

76.	de Orleans Casagrande P, Coimbra DR, de Souza LC, Andrade A. Effects of yoga on depressive symptoms, anxiety, sleep quality, and mood in patients with rheumatic diseases: Systematic review and meta-analysis. PM & R : the journal of injury, function, and rehabilitation. 2023;15(7):899-915.

77.	Della Valle E, Palermi S, Aloe I, Marcantonio R, Spera R, Montagnani S, et al. Effectiveness of Workplace Yoga Interventions to Reduce Perceived Stress in Employees: A Systematic Review and Meta-Analysis. Journal of functional morphology and kinesiology. 2020;5(2).

78.	Demir Yildirim A, Gungor Satilmis I. The Effects of Yoga on Pregnancy, Stress, and Anxiety in Infertile Individuals: A Systematic Review. Holist Nurs Pract. 2022;36(5):275-83.

79.	Denham-Jones L, Gaskell L, Spence N, Tim P. A systematic review of the effectiveness of yoga on pain, physical function, and quality of life in older adults with chronic musculoskeletal conditions. Musculoskeletal care. 2022;20(1):47-73.

80.	Desveaux L, Lee A, Goldstein R, Brooks D. Yoga in the Management of Chronic Disease: A Systematic Review and Meta-analysis. Medical care. 2015;53(7):653-61.

81.	Dhali B, Chatterjee S, Das SS, Cruz MD. Effect of Yoga and Walking on Glycemic Control for the Management of Type 2 Diabetes: A Systematic Review and Meta-analysis. Journal of the ASEAN Federation of Endocrine Societies. 2023;38(2).

82.	Dhali B, Chatterjee S, Das SS, Cruz MD. Effect of Yoga on Insulin Resistance in Type 2 Diabetes: A Systematic Review and Meta-Analysis. Clinical Diabetology. 2023;12(3):201-8.

83.	Djalilova DM, Schulz PS, Berger AM, Case AJ, Kupzyk KA, Ross AC. Impact of Yoga on Inflammatory Biomarkers: A Systematic Review. Biological research for nursing. 2019;21(2):198-209.

84.	Dong BXCJXLLTL. Yoga has a solid effect on cancer-related fatigue in patients with breast cancer: a meta-analysis. Breast Cancer Res Treat. 2019;177(1):5-16.

85.	Dunne EMBBLDMLFMMDJCDGS-BEWRRC. The benefits of yoga for people living with HIV/AIDS: A systematic review and meta-analysis. Complement Ther Clin Pract. 2019;34:157-64.

86.	Dutta A, Mooventhan A, Nivethitha L. Yoga as Adjunct Therapy for Chronic Heart Failure: A Systematic Review and Meta-Analysis of Randomized Controlled Trials. Avicenna journal of medicine. 2023;13(3):151-62.

87.	Dutta D, Bhattacharya S, Sharma M, Khandelwal D, Surana V, Kalra S. Effect of yoga on glycemia and lipid parameters in type-2 diabetes: a meta-analysis. Journal of diabetes and metabolic disorders. 2021;20(1):349-67.

88.	Falkenberg RIECPML. Yoga and immune system functioning: a systematic review of randomized controlled trials. J Behav Med. 2018;41(4):467-82.

89.	Feifei W, Szabo A. Effects of Yoga on Stress Among Healthy Adults: A Systematic Review. Alternative Therapies in Health & Medicine. 2020;26(4):58-64.

90.	Felbel S, Meerpohl JJ, Monsef I, Engert A, Skoetz N. Yoga in addition to standard care for patients with haematological malignancies. The Cochrane database of systematic reviews. 2014(6):CD010146.

91.	Ferreira-Vorkapic C, Feitoza JM, Marchioro M, Simoes J, Kozasa E, Telles S. Are There Benefits from Teaching Yoga at Schools? A Systematic Review of Randomized Control Trials of Yoga-Based Interventions. Evidence-based complementary and alternative medicine : eCAM. 2015;2015:345835.

92.	Francielly Silva D, Rodrigues Alves Santos ME, Silva do Carmo T, da Cruz Moura ME, e Mello Albuquerque Arruda IPdD, da Conceição Pereira S. Yoga implications for preventing accidental falls in older adults: a systematic review. Fisioterapia em Movimento. 2022;35:1-10.

93.	Galantino ML, Galbavy R, Quinn L. Therapeutic effects of yoga for children: a systematic review of the literature. Pediatric physical therapy : the official publication of the Section on Pediatrics of the American Physical Therapy Association. 2008;20(1):66-80.

94.	Galliford M, Robinson S, Bridge P, Carmichael M. Salute to the sun: a new dawn in yoga therapy for breast cancer. Journal of medical radiation sciences. 2017.

95.	García-Muñoz C, Villar-Alises O, Rodríguez-Sánchez-Laulhé P, Matias-Soto J, Martinez-Calderon J. No effects were found in favor of Hatha or Iyengar yoga exercises for improving cancer-related fatigue, depression symptoms, or quality of life: a systematic review with meta-analysis and metaregression. Supportive Care in Cancer. 2024;32(1):1-18.

96.	Ghazvineh D, Daneshvar M, Basirat V, Daneshzad E. The Effect of Yoga on the Lipid Profile: A Systematic Review and Meta-Analysis of Randomized Clinical Trials. Frontiers in nutrition. 2022;9:942702.

97.	Gomes-Neto M, Rodrigues ES, Silva WM, Carvalho VO. Effects of yoga in patients with chronic heart failure: A meta-analysis. Arq Bras Cardiol. 2014;103(5):433-9.

98.	Gong H, Ni C, Shen X, Wu T, Jiang C. Yoga for prenatal depression: a systematic review and meta-analysis. BMC psychiatry. 2015;15:14.

99.	Gonzalez M, Pascoe MC, Yang G, de Manincor M, Grant S, Lacey J, et al. Yoga for depression and anxiety symptoms in people with cancer: A systematic review and meta-analysis. Psycho-oncology. 2021;30(8):1196-208.

100.	Gothe NP, McAuley E. Yoga and Cognition: A Meta-Analysis of Chronic and Acute Effects. Psychosomatic medicine. 2015;77(7):784-97.

101.	Green E, Huynh A, Broussard L, Zunker B, Matthews J, Hilton CL, et al. Systematic Review of Yoga and Balance: Effect on Adults With Neuromuscular Impairment. The American journal of occupational therapy : official publication of the American Occupational Therapy Association. 2019;73(1):7301205150p1-p11.

102.	Haaz S, Bartlett SJ. Yoga for arthritis: a scoping review. Rheum Dis Clin North Am. 2011;37(1):33-46.

103.	Hagins M, States R, Selfe T, Innes K. Effectiveness of yoga for hypertension: systematic review and meta-analysis. Evidence-based complementary and alternative medicine : eCAM. 2013;2013:649836.

104.	Harder H, Parlour L, Jenkins V. Randomised controlled trials of yoga interventions for women with breast cancer: a systematic literature review. Supportive care in cancer : official journal of the Multinational Association of Supportive Care in Cancer. 2012;20(12):3055-64.

105.	Hartley L, Dyakova M, Holmes J, Clarke A, Lee MS, Ernst E, et al. Yoga for the primary prevention of cardiovascular disease. The Cochrane database of systematic reviews. 2014(5):CD010072.

106.	Hendriks T. The effects of Sahaja Yoga meditation on mental health: a systematic review. J Complement Integr Med. 2018;15(3):30.

107.	Hendriks TdJJCH. The Effects of Yoga on Positive Mental Health Among Healthy Adults: A Systematic Review and Meta-Analysis. J Altern Complement Med. 2017;23(7):505-17.

108.	Hill C. Is yoga an effective treatment in the management of patients with chronic low back pain compared with other care modalities - a systematic review. Journal of complementary & integrative medicine. 2013;10.

109.	Hofmann SG, Andreoli G, Carpenter JK, Curtiss J. Effect of Hatha Yoga on Anxiety: A Meta-Analysis. Journal of evidence-based medicine. 2016.

110.	Holtzman S, Beggs RT. Yoga for chronic low back pain: a meta-analysis of randomized controlled trials. Pain research & management. 2013;18(5):267-72.

111.	Hou L, Wang J, Mao M, Zhang Z, Liu D, Gao S, et al. Effect of yoga on cancer-related fatigue in patients with breast cancer: A systematic review and meta-analysis. Medicine. 2024;103(1):e36468.

112.	Hoy S, Östh J, Pascoe M, Kandola A, Hallgren M. Effects of yoga-based interventions on cognitive function in healthy older adults: A systematic review of randomized controlled trials. Complementary therapies in medicine. 2021;58:102690.

113.	Hsueh EJ, Loh EW, Lin JJA, Tam KW. Effects of yoga on improving quality of life in patients with breast cancer: a meta-analysis of randomized controlled trials. Breast Cancer. 2021;28(2):264-76.

114.	Innes KE, Bourguignon C, Taylor AG. Risk indices associated with the insulin resistance syndrome, cardiovascular disease, and possible protection with yoga: a systematic review. J Am Board Fam Pract. 2005;18(6):491-519.

115.	Innes KE, Selfe TK. Yoga for Adults with Type 2 Diabetes: A Systematic Review of Controlled Trials. Journal of diabetes research. 2016;2016:6979370.

116.	Innes KE, Vincent HK. The influence of yoga-based programs on risk profiles in adults with type 2 diabetes mellitus: a systematic review. Evidence-based complementary and alternative medicine : eCAM. 2007;4(4):469-86.

117.	Isath A, Kanwal A, Virk HUH, Bandyopadhyay D, Wang Z, Kumar A, et al. The Effect of Yoga on Cardiovascular Disease Risk Factors: A Meta-Analysis. Current Problems in Cardiology. 2023;48(5) (no pagination)(101593).

118.	James-Palmer A, Anderson EZ, Zucker L, Kofman Y, Daneault JF. Yoga as an Intervention for the Reduction of Symptoms of Anxiety and Depression in Children and Adolescents: A Systematic Review. Frontiers in pediatrics. 2020;8:78.

119.	Jasmin MS. Effectiveness of Yoga Intervention for Chronic Neck Pain: A Systematic Literature Review. International Journal of Caring Sciences. 2019;12(2):1088-96.

120.	Jayawardena R, Ranasinghe P, Ranawaka H, Gamage N, Dissanayake D, Misra A. Exploring the Therapeutic Benefits of Pranayama (Yogic Breathing): A Systematic Review. International journal of yoga. 2020;13(2):99-110.

121.	Jayawardena RRPCTAPMMA. The benefits of yoga practice compared to physical exercise in the management of type 2 Diabetes Mellitus: A systematic review and meta-analysis. Diabetes Metab Syndr. 2018;12(5):795-805.

122.	Jeter PE, Nkodo AF, Moonaz SH, Dagnelie G. A systematic review of yoga for balance in a healthy population. Journal of alternative and complementary medicine (New York, NY). 2014;20(4):221-32.

123.	Jihong S, Tao W, Yujie W, Rong L, Sitian N, Litao Z, et al. The Effectiveness of Yoga on Cancer-Related Fatigue: A Systematic Review and Meta-Analysis. Oncology Nursing Forum. 2021;48(2):207-28.

124.	Kaje KC, Dsilva F, Sanal TS, Latha T, Kumar S, D'Souza C. Effect of Yoga Intervention on Inflammatory Biomarkers among Women with Breast Cancer - A Systematic Review. Indian journal of palliative care. 2023;29(3):223-33.

125.	Kaligal C, Kanthi A, Mahadevappa V, Deepeshwar S. Influence of Yoga on Cognitive Functions in Individuals with Mental Disorders: A Systematic Review of Randomized Controlled Trials. Integrative & Complementary Therapies. 2023;29(1):15-24.

126.	Kalra S, Miraj M, Ajmera P, Shaik RA, Seyam MK, Shawky GM, et al. Effects of Yogic Interventions on Patients Diagnosed With Cardiac Diseases. A Systematic Review and Meta-Analysis. Frontiers in Cardiovascular Medicine. 2022;9(no pagination)(942740).

127.	Kan L, Zhang J, Yang Y, Wang P. The Effects of Yoga on Pain, Mobility, and Quality of Life in Patients with Knee Osteoarthritis: A Systematic Review. Evidence-based complementary and alternative medicine : eCAM. 2016;2016:6016532.

128.	Karamacoska D, Tan T, Mathersul DC, Sabag A, de Manincor M, Chang D, et al. A systematic review of the health effects of yoga for people with mild cognitive impairment and dementia. BMC geriatrics. 2023;23(1):37.

129.	Khajuria A, Kumar A, Joshi D, Kumaran SS. Reducing Stress with Yoga: A Systematic Review Based on Multimodal Biosignals. International journal of yoga. 2023;16(3):156-70.

130.	Khandekar JS, Vasavi VL, Singh VP, Samuel SR, Sudhan SG, Khandelwal B. Effect of Yoga on Blood Pressure in Prehypertension: A Systematic Review and Meta-Analysis. Scientific World Journal. 2021;2021(no pagination)(4039364).

131.	Khunti K, Boniface S, Norris E, De Oliveira CM, Nicola S. The effects of yoga on mental health in school-aged children: A Systematic Review and Narrative Synthesis of Randomised Control Trials. Clinical child psychology and psychiatry. 2023;28(3):1217-38.

132.	Kim KV, Bartley J, Ashe MC, Bardai Z, Butt DA, Chilibeck PD, et al. Effect of yoga on health-related outcomes in people at risk of fractures: a systematic review. Applied physiology, nutrition, and metabolism = Physiologie appliquee, nutrition et metabolisme. 2022;47(3):215-26.

133.	Kim S-D. Effects of yoga exercises for headaches: a systematic review of randomized controlled trials. J Phys Ther Sci. 2015;27(7):2377-80.

134.	Kim S-D. Effects of yoga on chronic neck pain: a systematic review of randomized controlled trials. J Phys Ther Sci. 2016;28(7):2171-4.

135.	Kim S-D. Twelve weeks of yoga for chronic nonspecific lower back pain: A meta-analysis. Pain Management Nursing. 2020;21(6):536-42.

136.	Kim SD. Psychological effects of yoga nidra in women with menstrual disorders: A systematic review of randomized controlled trials. Complement Ther Clin Pract. 2017;28:4-8.

137.	Kim SD. Yoga for menstrual pain in primary dysmenorrhea: A meta-analysis of randomized controlled trials. Complement Ther Clin Pract. 2019;36:94-9.

138.	Kirkwood G, Rampes H, Tuffrey V, Richardson J, Pilkington K. Yoga for anxiety: a systematic review of the research evidence. British journal of sports medicine. 2005;39(12):884-91; discussion 91.

139.	Kizhakkeveettil AWJSLHEL. Yoga for Quality of Life in Individuals With Chronic Disease: A Systematic Review. Altern Ther Health Med. 2019;25(1):36-43.

140.	Klatte R, Pabst S, Beelmann A, Rosendahl JS. The Efficacy of Body-Oriented Yoga in Mental Disorders. Deutsches Arzteblatt international. 2016;113(12):195-202.

141.	Klein J, Cook-Cottone C. The effects of yoga on eating disorder symptoms and correlates: a review. International journal of yoga therapy. 2013(23):41-50.

142.	Ko KY, Kwok ZCM, Chan HY. Effects of yoga on physical and psychological health among community-dwelling older adults: A systematic review and meta-analysis. International journal of older people nursing. 2023;18(5):e12562.

143.	Kongkaew CLPJKMPMPKKMP. The Effects of Thai Yoga on Physical Fitness: A Meta-Analysis of Randomized Control Trials. J Altern Complement Med. 2018;24(6):541-51.

144.	Kumar V, Jagannathan A, Philip M, Thulasi A, Angadi P, Raghuram N. Role of yoga for patients with type II diabetes mellitus: A systematic review and meta-analysis. Complement Ther Med. 2016;25:104-12.

145.	Kumari BNP, Shiny John KK, Manikya Latha J, Divya PD, Therese AM, Arulappan J. Effect of Yoga and Exercises to improve Physical Function and Quality of Life in Elderly: A Systematic Review of Randomized Controlled Trials. Indian Journal of Public Health Research and Development. 2022;13(3):327-31.

146.	Kuppusamy MKDPRAJSP. Effects of Bhramari Pranayama on health - A systematic review. J. 2018;8(1):11-6.

147.	Kuru Alici N, Arikan Dönmez A. A systematic review of the effect of laughter yoga on physical function and psychosocial outcomes in older adults. Complementary therapies in clinical practice. 2020;41:101252.

148.	Kwong Joey SW, Lau Hoi Lam C, Yeung F, Chau Pui H. Yoga for secondary prevention of coronary heart disease2015; (7). Available from: http://onlinelibrary.wiley.com/doi/10.1002/14651858.CD009506.pub4/abstract.

149.	Lauche R, Hunter DJ, Adams J, Cramer H. Yoga for Osteoarthritis: a Systematic Review and Meta-analysis. Current Rheumatology Reports. 2019;21(9) (no pagination)(47).

150.	Lauche R, Langhorst J, Lee MS, Dobos G, Cramer H. A systematic review and meta-analysis on the effects of yoga on weight-related outcomes. Prev Med. 2015;87:213-32.

151.	Lawrence MCFTMHHSGLBJBJ. Yoga for stroke rehabilitation. Cochrane Database of Systematic Reviews. 2017;2017 (12) (no pagination)(CD011483).

152.	Lee MS, Kim JI, Ha JY, Boddy K, Ernst E. Yoga for menopausal symptoms: a systematic review. Menopause (New York, NY). 2009;16(3):602-8.

153.	Lenoir Dit Caron R, Coquart J, Gilliaux M. Effect of yoga on health-related quality of life in central nervous system disorders: A systematic review. Clinical rehabilitation. 2021;35(11):1530-43.

154.	Levenhagen K, Davies C, Perdomo M, Ryans K, Gilchrist L. Effect of Yoga Among Women at Risk and With Breast Cancer–Related Lymphedema: A Systematic Review. Rehabilitation Oncology. 2023;41(3):129-38.

155.	Li CLYJYXLHZ. Efficacy of yoga training in chronic obstructive pulmonary disease patients: A systematic review and meta-analysis. Complement Ther Clin Pract. 2018;30:33-7.

156.	Li J, Gao X, Hao X, Kantas D, Mohamed EA, Zheng X, et al. Yoga for secondary prevention of coronary heart disease: A systematic review and meta-analysis. Complementary Therapies in Medicine. 2021;57(no pagination)(102643).

157.	Li YLSJJYS. Effects of yoga on patients with chronic nonspecific neck pain: A PRISMA systematic review and meta-analysis. Medicine (Baltimore). 2019;98(8):e14649.

158.	Lin KY, Hu YT, Chang KJ, Lin HF, Tsauo JY. Effects of yoga on psychological health, quality of life, and physical health of patients with cancer: a meta-analysis. Evidence-based complementary and alternative medicine : eCAM. 2011;2011:659876.

159.	Liu XC, Pan L, Hu Q, Dong WP, Yan JH, Dong L. Effects of yoga training in patients with chronic obstructive pulmonary disease: a systematic review and meta-analysis. Journal of thoracic disease. 2014;6(6):795-802.

160.	Loewenthal J, Innes KE, Mitzner M, Mita C, Orkaby AR. Effect of Yoga on Frailty in Older Adults : A Systematic Review. Annals of internal medicine. 2023;176(4):524-35.

161.	Long C, Ye J, Chen M, Gao D, Huang Q. Effectiveness of yoga therapy for migraine treatment: A meta-analysis of randomized controlled studies. The American journal of emergency medicine. 2022;58:95-9.

162.	Long Z, Shaoshuai W. The Efficacy of Prenatal Yoga on Labor Pain: A Systematic Review and Meta-analysis. Alternative Therapies in Health & Medicine. 2023;29(5):121-5.

163.	Lucovnik M, Pravst T, Dinevski IV, Zebeljan I, Dinevski D. Yoga during pregnancy: A systematic reviewJoga v nosecnosti: Sistematicni pregled literature. Zdravniski Vestnik. 2021;90(3-4):150-8.

164.	Luu K, Hall PA. Hatha Yoga and Executive Function: A Systematic Review. J Altern Complement Med. 2016;22(2):125-33.

165.	Ma X, Li SN, Chan DNS. Effects of Yoga on Cancer-Related Fatigue, Psychological Distress, and Quality of Life Among Patients With Cancer Undergoing Chemotherapy and/or Radiotherapy: A Systematic Review and Meta-analysis. Cancer nursing. 2023.

166.	Machado YDC, Oliveira M, Lima JLF, Bhargav H, Varambally S, de Miranda DM, et al. Effects of yoga on impulsivity in patients with and without mental disorders: a systematic review. BMC Psychiatry. 2024;24(1) (no pagination)(267).

167.	Mandlik GV, Siopis G, Nguyen B, Ding D, Edwards KM. Effect of a single session of yoga and meditation on stress reactivity: A systematic review. Stress and health : journal of the International Society for the Investigation of Stress. 2023.

168.	Martis CS, Chandrababu R, Ravishankar N, Bhandary RP, Mohammed CA, Tolson D, et al. The effectiveness of yoga therapy on caregivers of people living with dementia: A systematic review and meta-analysis of randomized controlled trials. Clinical Epidemiology and Global Health. 2023;19(no pagination)(101192).

169.	McGovern CECC. Yoga and Quality of Life in Women with Primary Dysmenorrhea: A Systematic Review. J Midwifery Womens Health. 2018;63(4):470-82.

170.	Miao C, Gao Y, Li X, Zhou Y, Chung JW, Smith GD. The effectiveness of mindfulness yoga on patients with major depressive disorder: a systematic review and meta-analysis of randomized controlled trials. BMC complementary medicine and therapies. 2023;23(1):313.

171.	Mockel L. Potential benefits of yoga in patients with heart failure: A meta-analysis of controlled trials. European Journal of Integrative Medicine. 2019;31 (no pagination)(100948).

172.	Munns L, Spark N, Crossland A, Preston C. The effects of yoga-based interventions on postnatal mental health and well-being: A systematic review. Heliyon. 2024;10(3):e25455.

173.	Nalbant G, Hassanein ZM, Lewis S, Chattopadhyay K. Content, Structure, and Delivery Characteristics of Yoga Interventions for Managing Hypertension: A Systematic Review and Meta-Analysis of Randomized Controlled Trials. Frontiers in public health. 2022;10:846231.

174.	Nawale A, Satya Lakshmi K, Sathyanath D, Muralidharan S. A Systematic Review on Effectiveness of Rajyoga Meditation on Chronic Tension Type Headache. Journal of Pharmaceutical Negative Results. 2022;13:1622-8.

175.	Ng QVNLWYWSLDCHSWS. A meta-analysis of the effectiveness of yoga-based interventions for maternal depression during pregnancy. Complementary Therapies in Clinical Practice. 2019;34:8-12.

176.	Nguyen-Feng VNCCJBME. Yoga as an intervention for psychological symptoms following trauma: A systematic review and quantitative synthesis. Psychol Serv. 2019;16(3):513-23.

177.	Niu N, Huang R, Zhao J, Zeng Y. Health benefits of yoga for cancer survivors: An updated systematic review and meta-analysis. Asia-Pacific journal of oncology nursing. 2024;11(3):100316.

178.	O'Neill M, Samaroo D, Lopez C, Tomlinson G, Santa Mina D, Sabiston C, et al. The Effect of Yoga Interventions on Cancer-Related Fatigue and Quality of Life for Women with Breast Cancer: A Systematic Review and Meta-Analysis of Randomized Controlled Trials. Integrative cancer therapies. 2020;19:1534735420959882.

179.	Oates J. The Effect of Yoga on Menstrual Disorders: A Systematic Review. J Altern Complement Med. 2017;23(6):407-17.

180.	Ostermann TVHBKCH. Effects of yoga on eating disorders-A systematic review. Complement Ther Med. 2019;46:73-80.

181.	Pal A, Nath B, Paul S, Meena S. Evaluation of the effectiveness of yoga in management of premenstrual syndrome: a systematic review and meta-analysis. Journal of Psychosomatic Obstetrics and Gynecology. 2022;43(4):517-25.

182.	Pal RGN. Yogic practices on oxidative stress and of antioxidant level: a systematic review of randomized controlled trials. J Complement Integr Med. 2017;25:25.

183.	Pan YYKWYZLLH. Could yoga practice improve treatment-related side effects and quality of life for women with breast cancer? A systematic review and meta-analysis. Asia Pac J Clin Oncol. 2017;13(2):e79-e95.

184.	Panebianco MSKRS. Yoga for epilepsy. Cochrane Database Syst Rev. 2017;10:CD001524.

185.	Pascoe MCTDRSCF. Yoga, mindfulness-based stress reduction and stress-related physiological measures: A meta-analysis. Psychoneuroendocrinology. 2017;86:152-68.

186.	Patel NK, Newstead AH, Ferrer RL. The effects of yoga on physical functioning and health related quality of life in older adults: a systematic review and meta-analysis. Journal of alternative and complementary medicine (New York, NY). 2012;18(10):902-17.

187.	Patil S, Biradar M, Khode V, Vadiraja H, Patil N, Raghavendra R. Effectiveness of yoga on arterial stiffness: A systematic review. Complementary Therapies in Medicine. 2020;52.

188.	Patil SG, Khode V, Christa E, Desai RM, Chandrasekaran AM, Vadiraja HS, et al. Effect of Yoga on Endothelial Function: A Systematic Review and Meta-Analysis. Journal of integrative and complementary medicine. 2024;30(3):233-49.

189.	Pilkington K, Kirkwood G, Rampes H, Richardson J. Yoga for depression: the research evidence. Journal of affective disorders. 2005;89(1-3):13-24.

190.	Posadzki P, Choi J, Lee MS, Ernst E. Yoga for addictions: A systematic review of randomised clinical trials. Focus on Alternative and Complementary Therapies. 2014;19(1):1-8.

191.	Posadzki P, Cramer H, Kuzdzal A, Lee MS, Ernst E. Yoga for hypertension: a systematic review of randomized clinical trials. Complementary therapies in medicine. 2014;22(3):511-22.

192.	Posadzki P, Ernst E. Yoga for low back pain: a systematic review of randomized clinical trials. Clinical rheumatology. 2011;30(9):1257-62.

193.	Posadzki P, Ernst E. Yoga for asthma? A systematic review of randomized clinical trials. The Journal of asthma : official journal of the Association for the Care of Asthma. 2011;48(6):632-9.

194.	Posadzki P, Ernst E, Terry R, Lee MS. Is yoga effective for pain? A systematic review of randomized clinical trials. Complementary therapies in medicine. 2011;19(5):281-7.

195.	Posadzki P, Kuzdzal A, Lee MS, Ernst E. Yoga for Heart Rate Variability: A Systematic Review and Meta-analysis of Randomized Clinical Trials. Applied psychophysiology and biofeedback. 2015;40(3):239-49.

196.	Puerto Valencia LMWASHBRSAHSHC. Yoga in the workplace and health outcomes: a systematic review. Occup Med (Oxf). 2019;69(3):195-203.

197.	Ramamoorthi R, Gahreman D, Skinner T, Moss S. The effect of yoga practice on glycemic control and other health parameters in the prediabetic state: A systematic review and meta-analysis. PLoS ONE. 2019;14(10) (no pagination)(e0221067).

198.	Rong L, Dai L-J, Ouyang Y-Q. The effectiveness of prenatal yoga on delivery outcomes: A meta-analysis. Complementary Therapies in Clinical Practice. 2020;39:N.PAG-N.PAG.

199.	Russell N, Daniels B, Smoot B, Allen DD. Effects of Yoga on Quality of Life and Pain in Women With Chronic Pelvic Pain: Systematic Review and Meta-Analysis. Journal of Women's Health Physical Therapy. 2019;43(3):144-54.

200.	Sadja J, Mills PJ. Effects of yoga interventions on fatigue in cancer patients and survivors: a systematic review of randomized controlled trials. Explore (New York, NY). 2013;9(4):232-43.

201.	Saraswathi V, Latha S, Niraimathi K, Vidhubala E. Managing Lymphedema, Increasing Range of Motion, and Quality of Life through Yoga Therapy among Breast Cancer Survivors: A Systematic Review. International journal of yoga. 2021;14(1):3-17.

202.	Schumann DADLRDGLJCH. Effect of Yoga in the Therapy of Irritable Bowel Syndrome: A Systematic Review. Clinical gastroenterology and hepatology : the official clinical practice journal of the American Gastroenterological Association. 2016;14(12):1720-31.

203.	Selvan P, Hriso C, Mitchell J, Newberg A. Systematic review of yoga for symptom management during conventional treatment of breast cancer patients. Complementary therapies in clinical practice. 2022;48:101581.

204.	Serwacki ML, Cook-Cottone C. Yoga in the schools: a systematic review of the literature. International journal of yoga therapy. 2012(22):101-9.

205.	Sharifi N, Afshari F, Bahri N. The effects of yoga on quality of life among postmenopausal women: A systematic review study. Post Reproductive Health. 2021;27(4):215-21.

206.	Sharma G, Mooventhan A, Naik G, Nivethitha L. A Review on Role of Yoga in the Management of Patients with Cardiac Arrhythmias. International journal of yoga. 2021;14(1):26-35.

207.	Sheffield KM, Woods-Giscombe CL. Efficacy, Feasibility, and Acceptability of Perinatal Yoga on Women's Mental Health and Well-Being: A Systematic Literature Review. Journal of holistic nursing : official journal of the American Holistic Nurses' Association. 2016;34(1):64-79.

208.	Shohani M, Kazemi F, Rahmati S, Azami M. The effect of yoga on the quality of life and fatigue in patients with multiple sclerosis: A systematic review and meta-analysis of randomized clinical trials. Complementary therapies in clinical practice. 2020;39:101087.

209.	Sieczkowska SMCPOCDRVGTALVAA. Effect of yoga on the quality of life of patients with rheumatic diseases: Systematic review with meta-analysis. Complement Ther Med. 2019;46:9-18.

210.	Silveira K, Smart CM. Cognitive, physical, and psychological benefits of yoga for acquired brain injuries: A systematic review of recent findings. Neuropsychological rehabilitation. 2020;30(7):1388-407.

211.	Sivaramakrishnan D, Fitzsimons C, Kelly P, Ludwig K, Mutrie N, Saunders DH, et al. The effects of yoga compared to active and inactive controls on physical function and health related quality of life in older adults- systematic review and meta-analysis of randomised controlled trials. International Journal of Behavioral Nutrition and Physical Activity. 2019;16(1) (no pagination)(33).

212.	Smith KB, Pukall CF. An evidence-based review of yoga as a complementary intervention for patients with cancer. Psycho-oncology. 2009;18(5):465-75.

213.	Suarez-Iglesias D, Santos L, Sanchez-Lastra MA, Ayan C. Systematic review and meta-analysis of randomised controlled trials on the effects of yoga in people with Parkinson's disease. Disability and rehabilitation. 2022;44(21):6210-29.

214.	Thayabaranathan TANEIMAHSSPSRKMCDA. Determining the potential benefits of yoga in chronic stroke care: a systematic review and meta-analysis. Topics in stroke rehabilitation. 2017:1-10.

215.	Thind HLRBBLDMLS-BEBBCS-SLAJ. The effects of yoga among adults with type 2 diabetes: A systematic review and meta-analysis. Prev Med. 2017;105:116-26.

216.	Tolia MTNNMMENIPAHJTKSDCNKDKVP-. Utilizing Yoga in Oncologic Patients Treated with Radiotherapy: Review. Indian J. 2018;24(3):355-8.

217.	Tulloch ABHDCTA. Yoga-based exercise improves health-related quality of life and mental well-being in older people: a systematic review of randomised controlled trials. Age Ageing. 2018;47(4):537-44.

218.	Tyagi A, Cohen M. Yoga and heart rate variability: A comprehensive review of the literature. International journal of yoga. 2016;9(2):97-113.

219.	Vancampfort D, Vansteelandt K, Scheewe T, Probst M, Knapen J, De Herdt A, et al. Yoga in schizophrenia: a systematic review of randomised controlled trials. Acta psychiatrica Scandinavica. 2012;126(1):12-20.

220.	Veneri DGMBMFHSE. Using the International Classification of Functioning, Disability, and Health Model to Gain Perspective of the Benefits of Yoga in Stroke, Multiple Sclerosis, and Children to Inform Practice for Children with Cerebral Palsy: A Meta-Analysis. J Altern Complement Med. 2018;24(5):439-57.

221.	Venugopal V, Geethanjali S, Poonguzhali S, Padmavathi R, Mahadevan S, Silambanan S, et al. Effect of Yoga on Oxidative Stress in Type 2 Diabetes Mellitus: A Systematic Review and Meta-analysis. Current diabetes reviews. 2022;18(2):e050421192663.

222.	Verma A, Upadhyay V, Saxena V. Effect of Yoga Therapy on Health Outcomes in Women With Polycystic Ovary Syndrome: A Systematic Review and Meta-Analysis. American journal of lifestyle medicine. 2023;17(1):73-92.

223.	Verzili B, Valerio de Arruda M, Herrmann F, Reyes MB, Galduroz RF. A systematic review with meta-analysis of Yoga's contributions to neuropsychiatric aspects of aging. Behavioural Brain Research. 2023;454(no pagination)(114636).

224.	Vizcaino M, Stover E. The effect of yoga practice on glycemic control and other health parameters in Type 2 diabetes mellitus patients: A systematic review and meta-analysis. Complementary therapies in medicine. 2016;28:57-66.

225.	Vollbehr NKB-VAANMHCSSLAHHJROBD. Hatha yoga for acute, chronic and/or treatment-resistant mood and anxiety disorders: A systematic review and meta-analysis. PLoS ONE. 2018;13(10):e0204925.

226.	Walia N, Matas J, Turner A, Gonzalez S, Zoorob R. Yoga for substance use: A systematic review. Journal of the American Board of Family Medicine. 2021;34(5):964-73.

227.	Wang G, Liang C, Sun G. Yoga's Therapeutic Effect on Perinatal Depression: A Systematic Review and Meta-Analysis. Psychiatria Danubina. 2022;34(2):195-204.

228.	Wang J, Xiong X, Liu W. Yoga for essential hypertension: a systematic review. PloS one. 2013;8(10):e76357.

229.	Wang WL, Chen KH, Pan YC, Yang SN, Chan YY. The effect of yoga on sleep quality and insomnia in women with sleep problems: A systematic review and meta-analysis. BMC Psychiatry. 2020;20(1) (no pagination)(195).

230.	Wang YLSWRJPRFWBZYHYZJ. Integrative effect of yoga practice in patients with knee arthritis: A PRISMA-compliant meta-analysis. Medicine (Baltimore). 2018;97(31):e11742.

231.	Ward L, Stebbings S, Cherkin D, Baxter GD. Yoga for functional ability, pain and psychosocial outcomes in musculoskeletal conditions: a systematic review and meta-analysis. Musculoskeletal care. 2013;11(4):203-17.

232.	Weaver LL, Darragh AR. Systematic Review of Yoga Interventions for Anxiety Reduction Among Children and Adolescents. The American journal of occupational therapy : official publication of the American Occupational Therapy Association. 2015;69(6):6906180070p1-9.

233.	Wei CWWYCCPYCPECCCTTH. Effectiveness of Yoga Interventions in Breast Cancer-Related lymphedema: A systematic review. Complement Ther Clin Pract. 2019;36:49-55.

234.	Wibowo RA, Nuramalia R, Nurrahma HA, Oktariani E, Setiawan J, Icanervilia AV, et al. The Effect of Yoga on Health-Related Fitness among Patients with Type 2 Diabetes Mellitus: A Systematic Review and Meta-Analysis. International Journal of Environmental Research and Public Health. 2022;19(7) (no pagination)(4199).

235.	Wieland LS, Skoetz N, Pilkington K, Harbin S, Vempati R, Berman BM. Yoga for chronic non-specific low back pain. Cochrane Database of Systematic Reviews. 2022;2022(11) (no pagination)(CD010671).

236.	Wieland LSSNLZSPSCDSN. Yoga for treating urinary incontinence in women. Cochrane Database Syst Rev. 2019;2:CD012668.

237.	Wu Y, Yan D, Yang J. Effectiveness of yoga for major depressive disorder: A systematic review and meta-analysis. Frontiers in Psychiatry. 2023;14(no pagination)(1138205).

238.	Wu YJBTARLCSLHKLJPCLPLS. Yoga as Antihypertensive Lifestyle Therapy: A Systematic Review and Meta-analysis. Mayo Clin Proc. 2019;94(3):432-46.

239.	Yang Y, Cao D, Lyu T, Gao W. Meta-analysis of a mindfulness yoga exercise intervention on depression - based on intervention studies in China. Frontiers in psychology. 2023;14:1283172.

240.	Yang ZY, Zhong HB, Mao C, Yuan JQ, Huang YF, Wu XY, et al. Yoga for asthma. The Cochrane database of systematic reviews. 2016;4:CD010346.

241.	Ye X, Chen Z, Shen Z, Chen G, Xu X. Yoga for Treating Rheumatoid Arthritis: A Systematic Review and Meta-Analysis. Frontiers in medicine. 2020;7:586665.

242.	Yeun Y-R, Kim S-D. Effects of yoga on immune function: A systematic review of randomized controlled trials. Complementary Therapies in Clinical Practice. 2021;44:N.PAG-N.PAG.

243.	Yi LJ, Tian X, Jin YF, Luo MJ, Jiménez-Herrera MF. Effects of yoga on health-related quality, physical health and psychological health in women with breast cancer receiving chemotherapy: a systematic review and meta-analysis. Annals of palliative medicine. 2021;10(2):1961-75.

244.	Yin J, Sun Y, Zhu Y, Alifujiang H, Wang Y, An S, et al. Effects of yoga on clinical symptoms, quality of life and social functioning in patients with schizophrenia: A systematic review and meta-analysis. Asian Journal of Psychiatry. 2024;93(no pagination)(103959).

245.	Youkhana S, Dean CM, Wolff M, Sherrington C, Tiedemann A. Yoga-based exercise improves balance and mobility in people aged 60 and over: a systematic review and meta-analysis. Age Ageing. 2016;45(1):21-9.

246.	Zhang J, Yang KH, Tian JH, Wang CM. Effects of yoga on psychologic function and quality of life in women with breast cancer: a meta-analysis of randomized controlled trials. Journal of alternative and complementary medicine (New York, NY). 2012;18(11):994-1002.

247.	Zhu F, Zhang M, Wang D, Hong Q, Zeng C, Chen W. Yoga compared to non-exercise or physical therapy exercise on pain, disability, and quality of life for patients with chronic low back pain: A systematic review and meta-analysis of randomized controlled trials. PloS one. 2020;15(9):e0238544.

248.	Zhu J, Chen X, Zhen X, Zheng H, Chen H, Liao H, et al. Meta-analysis of effects of yoga exercise intervention on sleep quality in breast cancer patients. Frontiers in oncology. 2023;13:1146433.

249.	Zoogman SGSBVEDMCML. Effect of yoga-based interventions for anxiety symptoms: A meta-analysis of randomized controlled trials. Spirituality in Clinical Practice. 2019;6(4):256-78.

250.	Zuo XLLQGFYLMFJ. Effects of yoga on negative emotions in patients with breast cancer: A meta-analysis of randomized controlled trials. International Journal of Nursing Sciences. 2016;3(3):299-306.
