# Supplementary material for: Evaluating Certainty of Evidence Assessment in Systematic Reviews of Yoga for Health: A Meta‐Research Study
Source: Cochrane Evid Synth Methods. 2026 Oct 31;4(6):e70110. doi: 10.1002/cesm.70110 (PMC13723278; doi:10.1002/cesm.70110)
Supplement: Supplementary file 3 [file CESM-4-e70110-s003.doc]

Appendix 2: Excluded studies

Not a systematic review (either clearly not or claims to be but does not meet criteria)

1. Bruce C, Achan V, Rathore S. Yoga-Based Cardiac Rehabilitation: Current Perspectives from Randomized Controlled Trials in Coronary Artery Disease. Vascular health and risk management. 2021;17:779-89.
2. Campanelli S, Tort ABL, Lobão-Soares B. Pranayamas and Their Neurophysiological Effects. International journal of yoga. 2020;13(3):183-92.
3. Chakrabarty J, Vidyasagar MS. Yoga as an Adjuvant for Cancer Patients in India. Forum of Clinical Oncology. 2021;11(3):17-22.
4. Eilat-Adar S, Shenhar M, Hellerstein D, Dunsky A. The Influence of Yoga on the Cognitive Function of People Aged 60 Years and Older: A Systematic Review. Alternative therapies in health and medicine. 2023;29(1):269-79.
5. El-Hashimi DGKM. Yoga-Specific Enhancement of Quality of Life Among Women With Breast Cancer: Systematic Review and Exploratory Meta-Analysis of Randomized Controlled Trials. J Evid Based Integr Med. 2019;24:2515690X19828325.
6. English A, McKibben E, Sivaramakrishnan D, Hart N, Richards J, Kelly P. A Rapid Review Exploring the Role of Yoga in Healing Psychological Trauma. International journal of environmental research and public health. 2022;19(23).
7. Ghuntla TP, Dholakiya JR. Analysis and Evaluation of Effectiveness of Yogic Activities on Blood Pressure Parameters of Hypertensive Patients in Indian Population: A Systematic Review and Meta-Analysis. International Journal of Pharmaceutical and Clinical Research. 2023;15(10):507-18.
8. Gothe NP, Khan I, Hayes J, Erlenbach E, Damoiseaux JS. Yoga Effects on Brain Health: A Systematic Review of the Current Literature. Brain Plasticity. 2019;5(1):105-22.
9. Hampton A, Bartz M. Therapeutic Efficacy of Yoga for Common Primary Care Conditions. WMJ : official publication of the State Medical Society of Wisconsin. 2021;120(4):293-300.
10. Jean M, Umair M, Muddaloor P, Farinango M, Ansary A, Dakka A, et al. The Effects of Yoga on Bipolar Disorder: A Systematic Review. Cureus. 2022;14(8):e27688.
11. KN H, Nilkantham S. Role of yoga in managing the consequences of work stress-a review. Health promotion international. 2023;38(3).
12. Kappas Mazzio A, Mendoza N, Lindsay Brown M, Sinha D, Messing J, Wilson S, et al. Yoga as a complementary approach to healing for adult victims and survivors of interpersonal violence. Complementary therapies in clinical practice. 2021;44:101427.
13. Kysar-Moon A, Vasquez M, Luppen T. Trauma-Sensitive Yoga Interventions and Posttraumatic Stress and Depression Outcomes Among Women: A Systematic Review and Analysis of Randomized Control Trials. International journal of yoga therapy. 2021;31(1).
14. Laplaud N, Perrochon A, Gallou-Guyot M, Moens M, Goudman L, David R, et al. Management of post-traumatic stress disorder symptoms by yoga: an overview. BMC complementary medicine and therapies. 2023;23(1):258.
15. Lin IH, Huang CY, Chou SH, Shih CL. Efficacy of Prenatal Yoga in the Treatment of Depression and Anxiety during Pregnancy: A Systematic Review and Meta-Analysis. International Journal of Environmental Research and Public Health. 2022;19(9) (no pagination)(5368).
16. Makone HB, Gawre VV, Hiray SA, Kulkarni GV. Effect of Bhramari Pranayam on cardiovascular, autonomic and central nervous system - A systematic review. Journal of Cardiovascular Disease Research. 2023;14(3):1236-44.
17. Miller S, Mendelson T, Lee-Winn A, Dyer NL, Khalsa SBS. Systematic review of randomized controlled trials testing the effects of yoga with youth. Mindfulness. 2020;11(6):1336-53.
18. Mooventhan A. A comprehensive review on scientific evidence-based effects (including adverse effects) of yoga for normal and high-risk pregnancy-related health problems. Journal of Bodywork and Movement Therapies. 2019;23(4):721-7.
19. Mooventhan A, Pavithra, Meha N, Monisha S, Deepika R, Poorani R, et al. A comprehensive scientific evidence-based impact of yoga in patients with rheumatoid arthritis. International Journal of Rheumatic Diseases. 2023;26(12):2359-65.
20. Nair J, Mishra A, Sharan AM. Assessment of the Impact of Yoga on the Quality of Life of Breast Cancer Patients: A Systematic Literature Review. Indian journal of palliative care. 2023;29(4):348-58.
21. Panjrath Y, Pathak VN. A Systematic Review on the Impact of Surya Namaskar and Loving Kindness Meditation in Treating Social Body Anxiety and Body Dissatisfaction in Women with Polycystic Ovarian Syndrome. Journal of Pharmaceutical Negative Results. 2022;13:10146-51.
22. Sagarwala R, Nasrallah HA. The effects of yoga on depression and motor function in patients with Parkinson's disease: A review of controlled studies. Annals of Clinical Psychiatry. 2020;32(3):209-15.
23. Shin S. Meta-analysis of the effect of yoga practice on physical fitness in the elderly. International Journal of Environmental Research and Public Health. 2021;18(21) (no pagination)(1663).
24. Singh A, Sethi J, Basavaraddi I. Yoga for Neck Pain: A Review. Alternative & Complementary Therapies. 2020;26(5):205-9.
25. Tamrakar S, Dudeja K, Kamble P, Suresh SB, Gupta S, Yogesh S, et al. Harmony of Mind and Body: Exploring the Impact of Yoga on Mental Health - a Systematic Review. International Journal of Academic Medicine and Pharmacy. 2023;5(6):387-90.
26. Tew GA, Ward L, Hewitt C, Tiedemann A. Does yoga reduce the risk of falls in older people? The BMJ. 2020;370(no pagination)(3246).
27. Thakur D, Saurabh SD, Tripathi D, Lufang D. Effect of yoga on polycystic ovarian syndrome: A systematic review. Journal of Bodywork and Movement Therapies. 2021;27:281-6.
28. Wilke E, Reindl W, Thomann P, Ebert M, Wuestenberg T, Thomann A. Effects of yoga in inflammatory bowel diseases and on frequent IBD-associated extraintestinal symptoms like fatigue and depression. Complementary Therapies in Clinical Practice. 2021;45.

Wrong publication format (e.g., abstract, summary)

1. Anheyer D, Haller H, Lauche R, Dobos G, Cramer PDH. Yoga for treating low back pain - a systematic review and meta-analysis. Advances in Integrative Medicine. 2019;6:S65-S.
2. Armer JS, Meis G, Lutgendorf SK. Can yoga practice improve fatigue in cancer survivorship? a meta-analysis. Psychosomatic Medicine. 2019;81(4):A35-A6.
3. Borden A, Cook-Cottone C. Yoga and eating disorder prevention and treatment: A comprehensive review and meta-analysis. New York, NY: Routledge; 2023. p. 97-134.
4. Bovas B. Effects of yoga therapy in migraine: A meta analysis. Cephalalgia. 2019;39(1 Supplement):249-50.
5. Cramer H, Anheyer D, Haller H, Lauche R. Yoga for low back pain: a systematic review and meta-analysis. European Journal of Integrative Medicine. 2021;Conference: ECIM 2022. Malaga Spain. 48(no pagination)(101956).
6. Ellis K, Wurz A, McLaughlin E, Schulte F, Sung L, Culos-Reed N. Yoga for children and adolescents affected by cancer or blood disease: Findings from a systematic review. Current Oncology. 2021;28(4):2616.
7. Ferguson L, Morean D. The Effects of Yoga Practices on Self-Regulation in Children with Autism Spectrum Disorder: A Systematic Review...National Institute on Disability, Independent Living, and Rehabilitation Research (NIDILRR) Advanced Rehabilitation Research and Training (AR. Archives of Physical Medicine & Rehabilitation. 2024;105(4):e157-e8.
8. Gonzalez M, Pascoe M, Yang G, De Manincor M, Grant S, Lacey J, et al. Yoga for depression and anxiety symptoms in people with cancer: A systematic review and meta-analysis. Supportive Care in Cancer. 2021;29(SUPPL 1):S208.
9. Haessel B, Pituskin E, McDermott S, Van Damme A, Norris C. The Effect of Yoga on Women's Cardiovascular Health: A Systematic Review. Canadian Journal of Cardiology. 2023;39(5 Supplement):S16.
10. Kowdley G, Castro P, Kowdley H, Ramji S, Joyner R. SKY and its effects on sleep: a systematic review of a breathing technique for sleep improvement. Sleep Medicine. 2024;115(Supplement 1):129.
11. Kowdley H, Kowdley G, Castro P, Hameem Z, Daquila D, Joyner R. Sudarshan Kriya Yoga (SKY) and its effects on stress/anxiety/well-being: a systematic review. Sleep Medicine. 2024;115:N.PAG-N.PAG.
12. Pathare N, Adams B, DiVirgilio CE. Does yoga therapy improve outcomes in individuals with congestive heart failure?: A systematic review. Cardiopulmonary Physical Therapy Journal. 2022;33(1):e24.
13. Patil SG, Khode V, Chandrasekaran AM, Desai RM, Vadiraja HS, Raghavendra R, et al. A systematic review and meta-analysis of effect of yoga and meditation on endothelial system. European Heart Journal. 2021;42(SUPPL 1):2444.
14. Pintaningrum Y, Dewi MK. Effect of Doing Yoga for 1 Month to Systolic and Diastolic Blood Pressure: A System Overview and Meta Analysis. Journal of Hypertension. 2023;41(Supplement 2):e4.
15. Prabhakaran D, Chandrasekaran AM. Yoga for the prevention of cardiovascular disease. Nature Reviews Cardiology. 2020;17(9):536-7.
16. Schoenburg R, Loewenthal J, Orkaby AR, Innes K. The effect of yoga on the frailty syndrome in older adults: A systematic review. Journal of the American Geriatrics Society. 2021;69(SUPPL 1):S146.
17. Srinivas S, Ashraf S, Bachu A, Kim F, Shah K. Yoga as an Adjunct Treatment for Attention Deficit Hyperactivity Disorder (ADHD). European Psychiatry. 2023;66(Supplement 1):S727.
18. Verma A, Upadhyay V, Saxena V. Effect of yoga therapy on health of women with polycystic ovary syndrome: systematic-review and meta-analysis. International Journal of Epidemiology. 2021;50(Supplement 1):i242.

Not restricted to yoga as the intervention of interest

1. Acabchuk RL, Brisson JM, Park CL, Babbott-Bryan N, Parmelee OA, Johnson BT. Therapeutic Effects of Meditation, Yoga, and Mindfulness-Based Interventions for Chronic Symptoms of Mild Traumatic Brain Injury: A Systematic Review and Meta-Analysis. Applied psychology. 2021;Health and well-being. 13(1):34-62.
2. Tang MF, Chiu HY, Xu X, Kwok JY, Cheung DST, Chen CY, et al. Walking is more effective than yoga at reducing sleep disturbance in cancer patients: A systematic review and meta-analysis of randomized controlled trials. Sleep Med Rev. 2019;47:1-8.
3. Gonzalez NA, Sakhamuri N, Athiyaman S, Randhi B, Gutlapalli SD, Pu J, et al. A Systematic Review of Yoga and Meditation for Attention-Deficit/Hyperactivity Disorder in Children. Cureus. 2023;15(3):e36143.
4. Gunjawate DR, Ravi R. Effect of yoga and meditation on tinnitus: a systematic review. The Journal of laryngology and otology. 2021;135(4):284-7.
5. Gupta S, Jhanjee S, Dhawan A. Effectiveness of Interventions Based on Yogic Breathing Practices (IB-YBP) on Substance Use Disorders-A Systematic Review of the Randomized Control Trials and Quasi-Experimental Trials. Substance use & misuse. 2021;56(11):1624-41.
6. Jiang T, Hou J, Sun R, Dai L, Wang W, Wu H, et al. Immunological and Psychological Efficacy of Meditation/Yoga Intervention Among People Living With HIV (PLWH): A Systematic Review and Meta-analyses of 19 Randomized Controlled Trials. Annals of behavioral medicine : a publication of the Society of Behavioral Medicine. 2021;55(6):505-19.
7. Kanaujia S, Saraswati P, Anshu, Singh N, Singh S, Kataria N, et al. Effect of yoga and mindfulness on psychological correlates in young athletes: A meta-analysis. Journal of Ayurveda and integrative medicine. 2023;14(3):100725.
8. Ogden SL. A meta-analysis of trauma informed yoga programs for posttraumatic stress disorder in active-duty members and veterans: ProQuest Information & Learning; 2024.
9. Pareek R, Ojha N. Evidence based study of effect of Swarna Yoga (Gold formulations) in children: A systematic review. International Journal of Pharmaceutical Sciences and Research. 2021;12(12):6209-13.
10. Shi H, Wu Y, Wang L, Zhou X, Li F. Effects of Laughter Therapy on Improving Negative Emotions Associated with Cancer: A Systematic Review and Meta-Analysis. Oncology. 2024;102(4):343-53.
11. Shivangani J, Neelam B. Randomized Controlled Trial on the Effect of Yoga and Meditation on Cognitive Function: A Systematic Review & Metaanalysis. International Journal of Current Pharmaceutical Review and Research. 2022;14(4):50-9.
12. Sun Y, Lamoreau R, O'Connell S, Horlick R, Bazzano AN. Yoga and Mindfulness Interventions for Preschool-Aged Children in Educational Settings: A Systematic Review. International journal of environmental research and public health. 2021;18(11).
13. van der Wal CN, Kok RN. Laughter-inducing therapies: Systematic review and meta-analysis. Social Science & Medicine. 2019;232:473-88.
14. Yin J, Tang L, Dishman RK. The effects of a single session of mindful exercise on anxiety: A systematic review and meta-analysis. Mental Health and Physical Activity. 2021;21(no pagination)(100403).

Systematic review is only one part of a larger project

1. Burnout: Can yoga help you cope? IQWiG Reports – Commission No. HT21-02. Cologne Germany: © IQWiG (Institute for Quality and Efficiency in Health Care). 2024.
2. Holt MB. The impact of yoga programs for people who have experienced trauma: A systematic review: ProQuest Information & Learning; 2019.
3. Slosman K. Effects of yoga practice in individuals with mild to moderate Parkinson's disease for improving motor disability and balance: A Meta-Analysis: ProQuest Information & Learning; 2019.
4. Weaver LL. Yoga for anxiety reduction in children and adolescents: A mixed methods effectiveness study: ProQuest Information & Learning; 2019.

Not in English

1. Kang Y, Yu H, Xing T, Chen Z. Efficacy of yoga intervention for low back pain: A meta-analysis. [Chinese]Meta. Chinese Journal of Evidence-Based Medicine. 2020;20(1):47-52.
2. 章毛毛, 孟爱凤, 张柳柳, 鄴 萍, 智晓旭, 陆宁宁, et al. 不同方式瑜伽干预对乳腺癌病人睡眠质量 影响的Meta分析. Chinese Evidence-based Nursing. 2021;7(18):2415-22.

Not about treatment or prevention of health condition/symptom

1. Moeyaert M, Fingerhut J. Quantitative Synthesis of Personalized Trials Studies: Meta-Analysis of Aggregated Data Versus Individual Patient Data. Harvard data science review. 2022;4(SI3).
